# CSF production rate, resistance to reabsorption, and intracranial pressure: a systematic review and meta-analysis

**DOI:** 10.1093/braincomms/fcaf044

**Published:** 2025-01-30

**Authors:** Ihsane Olakorede, Stefan Y Bögli, Zofia Czosnyka, Marek Czosnyka, Peter Smielewski

**Affiliations:** Brain Physics Laboratory, Division of Neurosurgery, Department of Clinical Neurosciences, University of Cambridge, Cambridge CB2 0QQ, UK; Brain Physics Laboratory, Division of Neurosurgery, Department of Clinical Neurosciences, University of Cambridge, Cambridge CB2 0QQ, UK; Brain Physics Laboratory, Division of Neurosurgery, Department of Clinical Neurosciences, University of Cambridge, Cambridge CB2 0QQ, UK; Brain Physics Laboratory, Division of Neurosurgery, Department of Clinical Neurosciences, University of Cambridge, Cambridge CB2 0QQ, UK; Brain Physics Laboratory, Division of Neurosurgery, Department of Clinical Neurosciences, University of Cambridge, Cambridge CB2 0QQ, UK

**Keywords:** cerebrospinal fluid, intracranial hypertension, intracranial pressure, meta-analysis, systematic review

## Abstract

Davson’s equation relates the state of stable intracranial pressure (ICP) to the production rate of CSF (I_F_) and resistance to CSF outflow (R_OUT_). Both parameters are assumed to be independent of ICP, but results are conflicting. The objective is to define the relationship between ICP, I_F_ and R_OUT_ using a systematic literature review. Medline and Embase were searched from inception up to 12 February 2024. Experimental studies exploring the association between ICP, I_F,_ and R_OUT_ were included. Individual measurements of ICP, I_F_ and/or R_OUT_ were extracted from tables or graphs, alongside descriptive parameters (population, ICP measurement site, disease, and computational method). Linear regression and mixed effects models were applied. From 1304 references, 25 articles were included in our meta-analysis. I_F_ is approximately constant across all pathologies independent of the ICP level, population, disease, ICP measurement site and the measurement/estimation method. Conversely, ICP was positively correlated with R_OUT_. The intercorrelation, however, differed by population, disease, ICP measurement site and estimation method. Additionally, I_F_ derived from Davson’s Equation compared with the measured I_F_ were similar for patients with hydrocephalus but differed for patients with acute brain injury. Davson’s Equation describes the various components of cerebrospinal fluid dynamics. The results underline important caveats for its use in patients with acute brain injury wherein the estimated values differ from the measured ones. Overall, additional metrics describing the cerebrovascular system or the underlying disease have to be taken into account for more accurate estimations.

## Teaser

Olakorede *et al*. confirm constant cerebrospinal fluid production despite intracranial pressure variations, influenced by age and disease. They demonstrate increasing cerebrospinal fluid reabsorption resistance with pressure, greater in acute than chronic conditions. Their meta-analysis supports Davson’s equation for hydrocephalus but not other neurological disorders, emphasizing variations in cerebrospinal fluid dynamics across pathological states.

## Graphical Abstract

**graphical abstract:**
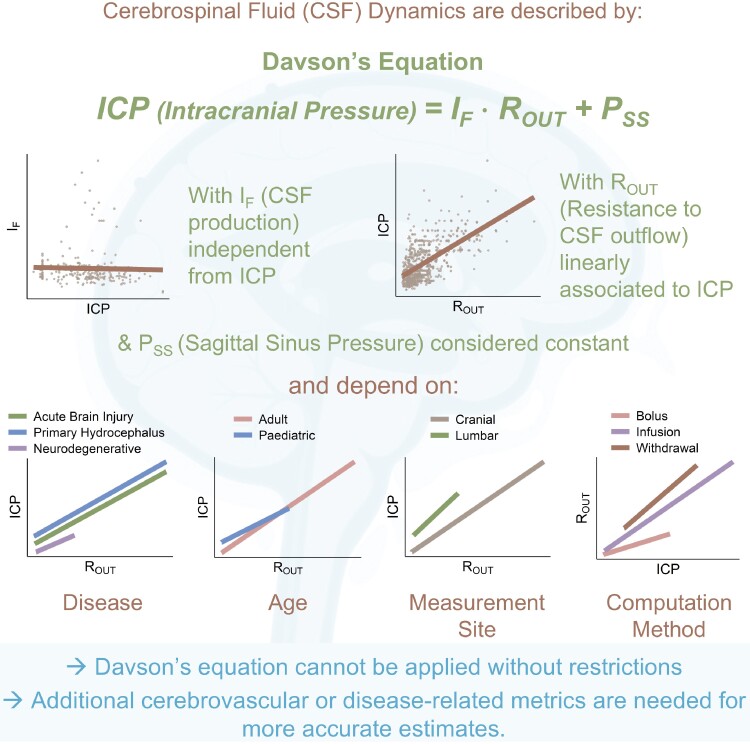

## Introduction

Intracranial pressure (ICP) refers to the pressure inside the skull, including the brain tissue, the vasculature and the cerebrospinal fluid (CSF) space. A non-compensated change in volume of either compartment, e.g. due to a severe acute brain injury (ABI; increase of tissue volume) or a hydrocephalus (increase in CSF volume) impacts ICP. The choroid plexus, located within the ventricles, was believed to be the sole site of CSF production. However, several studies have challenged this view, demonstrating the existence of alternative CSF production sites and mechanisms beyond the choroid plexus.^1,2^ CSF transports nutrients and acts as the brain's waste disposal system. In addition, CSF facilitates humoral communication between various brain regions and provides mechanical support for the brain.^3^

Various methods for CSF production rate (I_F_) quantification exist, including direct (via collection of CSF from the lateral ventricles) and indirect (acquisition of CSF from the cisterna magna) invasive procedures or non-invasive techniques that assess CSF flow using, for example magnetic resonance tomography.^4^ CSF is primarily reabsorbed into venous blood via the arachnoid granulations into the superior sagittal sinus in adults. The nature of CSF outflow is assumed to be linear. The reabsorption rate is implied to be proportional to the pressure gradient between the CSF (ICP) and sagittal sinus (P_SS_) pressures. The resistance to CSF outflow (R_OUT_) is defined as the inverse of the proportionality coefficient.^5^

Three invasive methods are employed to estimate R_OUT_: (i) Bolus, a very brief infusion of fluid into the CSF space resulting in an abrupt increase in ICP; (ii) Infusion, a prolonged, continuous infusion of fluid into the CSF space until a positive pressure plateau is reached; (iii) Withdrawal, a continuous removal of fluid from the CSF space, lowering ICP. Based on the changes in CSF volume and ICP, R_OUT_ is calculated.

ICP can be estimated based solely on the CSF compartment parameters, as described by Davson’s equation (Eq. 1).


(1) $$\mathrm{ICP}=\;R_{\mathrm{OUT}}\times I_{F}+P_{\mathrm{SS}}$$

Davson’s equation describes the CSF hydrodynamics by relating ICP to I_F_, R_OUT_, and P_SS_. P_SS_^6^ and I_F_^7^ are considered to be constant and independent of ICP, in spite of known rare exceptions. In idiopathic intracranial hypertension (IIH), P_SS_ is related to the ICP level^8^ potentially due to the increased downstream venous resistance. In the normal ICP range, R_OUT_ is believed to be pressure-independent.^9^ This equation has been widely applied to describe and diagnose CSF circulatory disorders and has been used within various models describing cerebrovascular states.

Whether these parameters are actually constant remains elusive with contradicting results from different studies. By performing a systematic review and meta-analysis, we aim to: (i) quantify the association between I_F_, R_OUT_ and ICP; (ii) assess whether other descriptives [e.g. patient characteristics (age, and in particular disease), measurement parameters including ICP measurement site or the choice of computation method] affect these relationships and (iii) assess whether Davson’s equation can be applied to the various diseases associated with changes in CSF dynamics. This could assist in further validating Davson's equation, clarify the way these parameters are associated with ICP, and dispel some of the hypotheses surrounding the CSF circulation system.

## Materials and methods

The protocol was registered (PROSPERO ID CRD42024523679) and the standards of the Preferred Reporting Items for Systematic Reviews and Meta-Analyses standards were followed. The Preferred Reporting Items for Systematic Reviews and Meta-Analyses checklist can be viewed in Supplementary Table 1.

### Information source and search strategy

From Inception to 12 February 2024, two databases—MEDLINE and EMBASE—were searched. Synonyms for ICP changes were combined with synonyms for CSF production and reabsorption as the overall strategy, presented in Supplementary Fig. 1.

### Study selection and data extraction

The only inclusion criteria were the availability of I_F_, R_OUT_, or both, alongside measured baseline ICP. The exclusion criteria: (i) specific publication formats (case studies, editorials/comments, reviews, and references solely accessible in an abstract format); (ii) studies without explicit description of I_F_ or R_OUT_; (iii) studies whose scope extended beyond the investigation of the dynamics between CSF and ICP; (iv) studies written in languages other than English and French; (v) studies involving a third factor—typically a pharmacological component—that directly affects the production or reabsorption of CSF; (vi) animal/preclinical studies; (vii) theoretical modelling studies and (viii) studies without a change in ICP.

Two investigators (IO, SB), blinded to each other's assessments, screened the titles and abstracts (Step 1) and full texts (Step 2) of each publication. Any disputes regarding inclusion were settled by arbitration and consensus among the review team members. Rayyan,^10^ a reference management system allowing for blinded screening, was used for eligibility screening. Collected data included first author, publication year, measured variables (I_F_, R_OUT_), cohort size, population (age <16 was considered as paediatric, adult otherwise), disease(s), ICP measurement site, type of intervention(s) (bolus, infusion or withdrawal), ICP range and computational method (formula used). Specifically, measurements were divided into four disease groups depending on the characteristics of the ICP change: ABI (traumatic brain injury, TBI; subarachnoid haemorrhage, SAH or others—acute increase in ICP), brain tumour (arteriovenous malformation, brain tumour—chronic increase in tissue or blood volume), neurodegenerative (brain atrophy, normal-pressure hydrocephalus, Parkinson's disease—tissue degeneration without increase in ICP) and primary hydrocephalus (craniosynostosis, IIH, high-pressure hydrocephalus, hydrocephalus—chronic increase in ICP). ICP measurement sites have been split into two categories: cranial site, extraventricular drain or parenchymal wire and lumbar site. Using WebPlotDigitizer, extraction of individual values of ICP, I_F_ and R_OUT_ was performed from tables and graphs.

### Bias appraisal

Quality assessment of included studies was performed using the National Institutes of Health Quality Assessment Tool of Case Series Studies.

### Statistical analysis

To determine the existence of a linear relationship between ICP, R_OUT_ and I_F_, linear regression models were applied to the whole dataset. For comparison purposes, all units were initially and appropriately converted to mmHg, mL and min. Four additional parameters were evaluated employing sub-group analyses: population, disease, ICP measurement site and intervention. Both the physiological relationship between ICP and R_OUT_ as specified by Davson's equation and the impact of R_OUT_ on ICP were examined. Furthermore, a one-sample, two-tailed Wilcoxon signed-rank test was performed to compare the I_F_ measurements performed in each studied sub-group to the I_F_ approximations corresponding to the slope of each ICP-R_OUT_ linear relationship. As part of a secondary analysis, two mixed effect models were applied to I_F_ and R_OUT_ with the individual research studies included as a random effect operating on the models’ intercept and all the previously mentioned factors included as fixed effects. The significance of these parameters was evaluated using Analysis of Variance (ANOVA). These analyses and figure preparations were carried out using the R V.4.4.1 packages ‘ggplot2,’ ‘rstatix,’ ‘lme4’ and ‘lmer.’

## Results

### Study selection output

**Figure 1:**
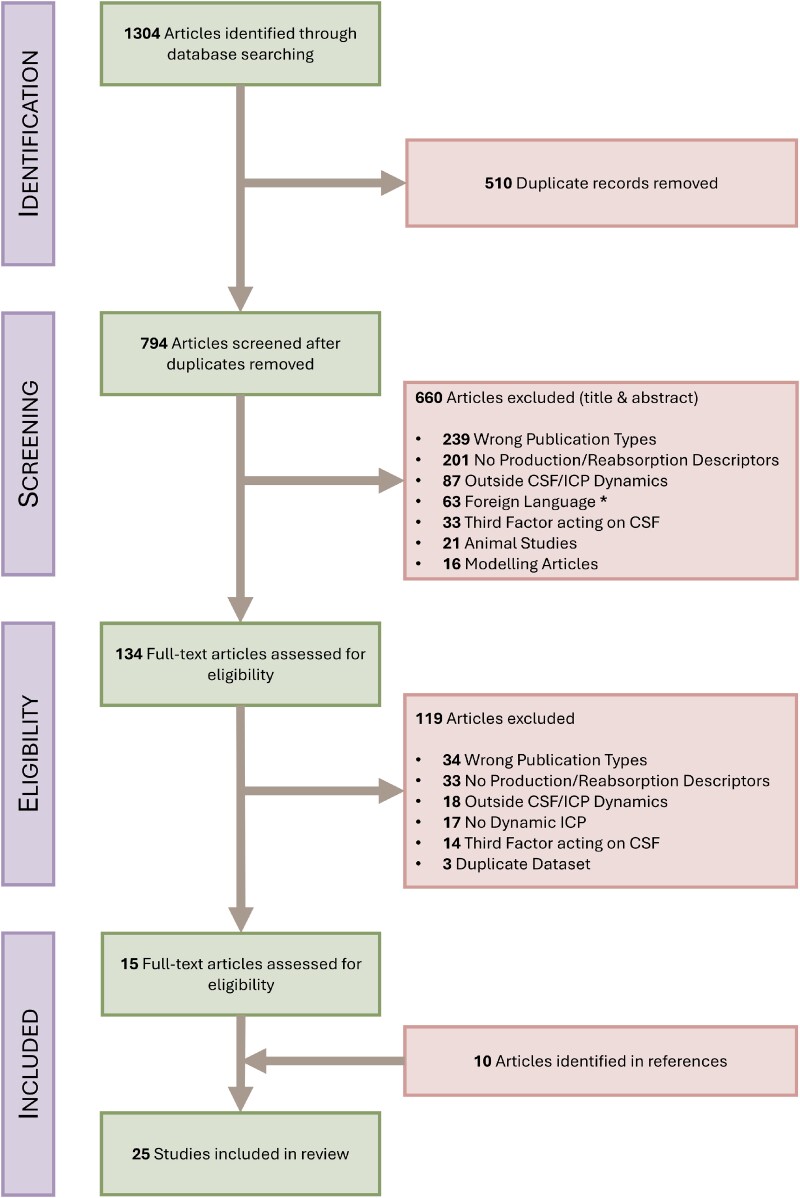
**Preferred Reporting Items for Systematic Reviews and Meta-Analyses flow diagram**. *Language of excluded studies: Japanese (25), German (13), Russian (12), Spanish (5), Polish (4), Italian (3), Chinese (1), Hungarian (1), Korean (1) and Turkish (1).

The study selection is shown in Fig. 1*(Preferred Reporting Items for Systematic Reviews and Meta-Analyses Flow Chart)*. Out of initially 1304 studies, a total of 25 studies were included in the final review. All studies were peer-reviewed journal articles, with half being published between 1980 and 1989.

### Summary of extracted articles

**Figure 2:**
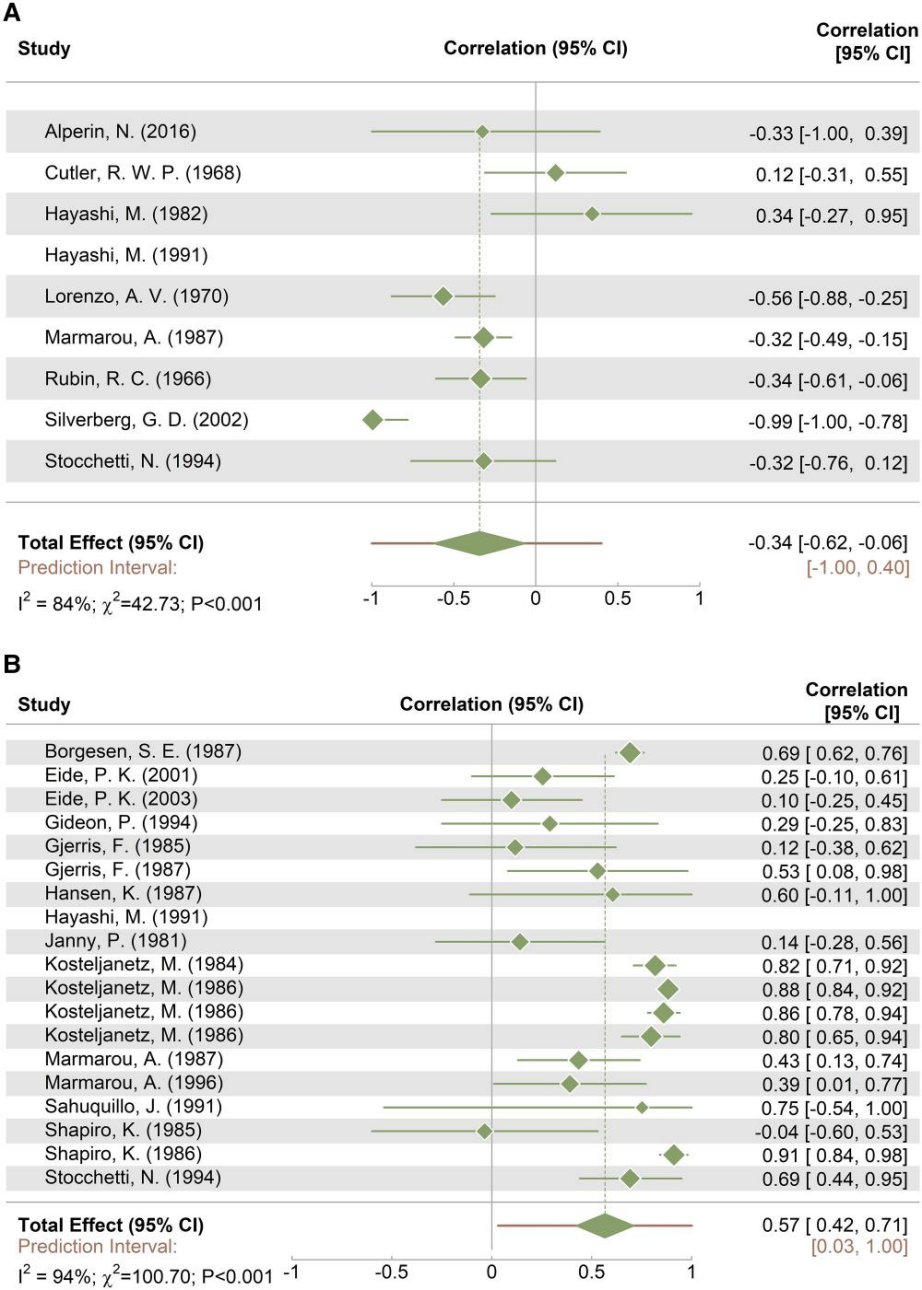
**Forest plot of the association of intracranial pressure (ICP) with CSF production rate (I_F_) and resistance to outflow (R_OUT_).** (**A**) I_F_ (*N* = 8), and (**B**) R_OUT_ (*N* = 18). Data name: studies; data type: continuous; effect measure: correlation; analysis model: mixed effects; statistical method: χ^2^ test. CI, confidence interval; *I*², heterogeneity measure; χ², value of χ² test for heterogeneity and *P*, statistical significance of the χ² test.

A summary of all eligible studies is presented in Tables 1 and 2, with comprehensive details available in Supplementary Tables 2 and 3. A bias appraisal was performed using the NIH Quality Assessment Tool (Supplementary Table 4), with 52% of the studies rated as ‘Good’ and 44% as ‘Fair’. A total of 243 individual I_F_ measurements and 676 R_OUT_ approximations were performed in 860 individuals (about 86% adult, no healthy subjects). The main diseases examined were hydrocephalus (15%) and ABI (27%). 45% of the measurements were classified as ‘mixed disease’ due to the lack of description for the individual patients. Only 10% of measurements were made in the lumbar area. In 50% of the cases, resistance to CSF outflow was assessed by infusion, 41% by bolus, and 9% by withdrawal. R_OUT_ computation methods, presented in Supplementary Table 5, were categorized based on the type of intervention used to approximate the resistance to CSF outflow. A forest plot summarizing the correlation between ICP, R_OUT_ and I_F_ of all included studies is shown in Fig. 2, alongside the results of the heterogeneity test and the prediction interval.

**Table 1:** Characteristics of 25 studies included in the meta-analysis

Ref	Author (year)	Variable	Population	Sex	Number of patients	Disease
^11^	Alperin, N. (2016)	I_F_	Adult	F	7	IIH
^12^	Borgesen, S. E. (1987)	R_OUT_	Adult	NR	230	HPH, IIH, NPH
^13^	Cutler, R. W. P. (1968)	I_F_	Paediatric	F, M	12	BT, cerebral infection
^14^	Eide, P. K. (2001)	R_OUT_	Paediatric	F, M	28	Craniosynostosis, hydrocephalus
^15^	Eide, P. K. (2003)	R_OUT_	Adult	F, M	16	NPH
^16^	Gideon, P. (1994)	R_OUT_	Adult, paediatric	F, M	12	IIH
^17^	Gjerris, F. (1985)	R_OUT_	Adult, paediatric	F, M	14	IIH
^18^	Gjerris, F. (1987)	R_OUT_	Adult	F, M	11	HPH, SAH
^19^	Hansen, K. (1987)	R_OUT_	Adult	F, M	4	Meningitis, spinal tumour
^20^	Hayashi, M. (1982)	I_F_	Adult	F, M	9	AS, BT, chronic SDH, IIH, NPH
^21^	Hayashi, M. (1991)	I_F_, R_OUT_	Adult	F, M	94	BT, hydrocephalus, intracranial haemorrhage, IIH, meningitis, SAH, superior sagittal sinus thrombosis
^22^	Janny, P. (1981)	R_OUT_	Adult, Paediatric	F, M	22	AVM, IIH, meningioma, meningitis
^23^	Kosteljanetz, M. (1984)	R_OUT_	Adult	F, M	17	SAH
^24^	Kosteljanetz, M. (1986)	R_OUT_	Adult	NR	62	Hydrocephalus, SAH, TBI
^25^	Kosteljanetz, M. (1986)	R_OUT_	Adult	F, M	16	TBI
^26^	Kosteljanetz, M. (1986)	R_OUT_	Adult	F, M	26	NPH
^27^	Lorenzo, A. V. (1970)	I_F_	Adult, Paediatric	F, M	12	AS, cerebral infection, hydrocephalus, meningitis, NPH
^28^	Marmarou, A. (1987)	I_F_, R_OUT_	Adult	NR	28	TBI
^29^	Marmarou, A. (1996)	R_OUT_	Adult	NR	75	Atrophy, HPH, IIH, NPH, TBI
^30^	Rubin, R. C. (1966)	I_F_	Adult	NR	11	BT
^31^	Sahuquillo, J. (1991)	R_OUT_	Adult	F, M	54	Hydrocephalus
^32^	Shapiro, K. (1985)	R_OUT_	Paediatric	NR	13	Hydrocephalus
^33^	Shapiro, K. (1986)	R_OUT_	Paediatric	NR	20	Hydrocephalus
^34^	Silverberg, G. D. (2002)	I_F_	Adult, paediatric	NR	30	Hydrocephalus, Parkinson
^35^	Stocchetti, N. (1994)	I_F_, R_OUT_	Adult	F, M	17	SAH

AS, aqueduct stenosis; AVM, arteriovenous malformation; BT, brain tumour; F, female; HPH, high-pressure hydrocephalus; IIH, idiopathic intracranial hypertension; M, male; NPH, normal-pressure hydrocephalus; NR, not reported; SAH, subarachnoid haemorrhage; TBI, traumatic brain injury.

**Table 2:** Methodological characteristics of included studies

Ref	Author (year)	Intervention	ICP measurement site	Methods I_F_/R_OUT_
^11^	Alperin, N. (2016)	Withdrawal	Lumbar puncture	1
^12^	Borgesen, S. E. (1987)	Infusion	EVD	4
^13^	Cutler, R. W. P. (1968)	Infusion	Lumbar puncture	2
^14^	Eide, P. K. (2001)	Infusion	Wire	7
^15^	Eide, P. K. (2003)	Infusion	EVD	7
^16^	Gideon, P. (1994)	Infusion	Lumbar puncture	Not provided
^17^	Gjerris, F. (1985)	Infusion	Lumbar puncture	4
^18^	Gjerris, F. (1987)	Infusion	EVD	4
^19^	Hansen, K. (1987)	Infusion	EVD, wire	4
^20^	Hayashi, M. (1982)	Withdrawal	EVD	1
^21^	Hayashi, M. (1991)	Infusion	EVD	1, 4
^22^	Janny, P. (1981)	Infusion	EVD	Not provided
^23^	Kosteljanetz, M. (1984)	Bolus, infusion, withdrawal	EVD	5, 7, 8
^24^	Kosteljanetz, M. (1986)	Bolus, infusion, withdrawal	EVD	5, 7, 8
^25^	Kosteljanetz, M. (1986)	Bolus, withdrawal	EVD	5, 8
^26^	Kosteljanetz, M. (1986)	Bolus	EVD	5
^27^	Lorenzo, A. V. (1970)	Infusion	Lumbar puncture	2
^28^	Marmarou, A. (1987)	Bolus, withdrawal	EVD	3, 5
^29^	Marmarou, A. (1996)	Bolus	Lumbar puncture	6
^30^	Rubin, R. C. (1966)	Infusion	EVD	2
^31^	Sahuquillo, J. (1991)	Bolus	Wire	5
^32^	Shapiro, K. (1985)	Bolus	EVD	6
^33^	Shapiro, K. (1986)	Bolus	EVD	6
^34^	Silverberg, G. D. (2002)	Withdrawal	EVD	1
^35^	Stocchetti, N. (1994)	Bolus, withdrawal	EVD	3, 5

EVD, extraventricular drain.

### Primary analysis

**Figure 3:**
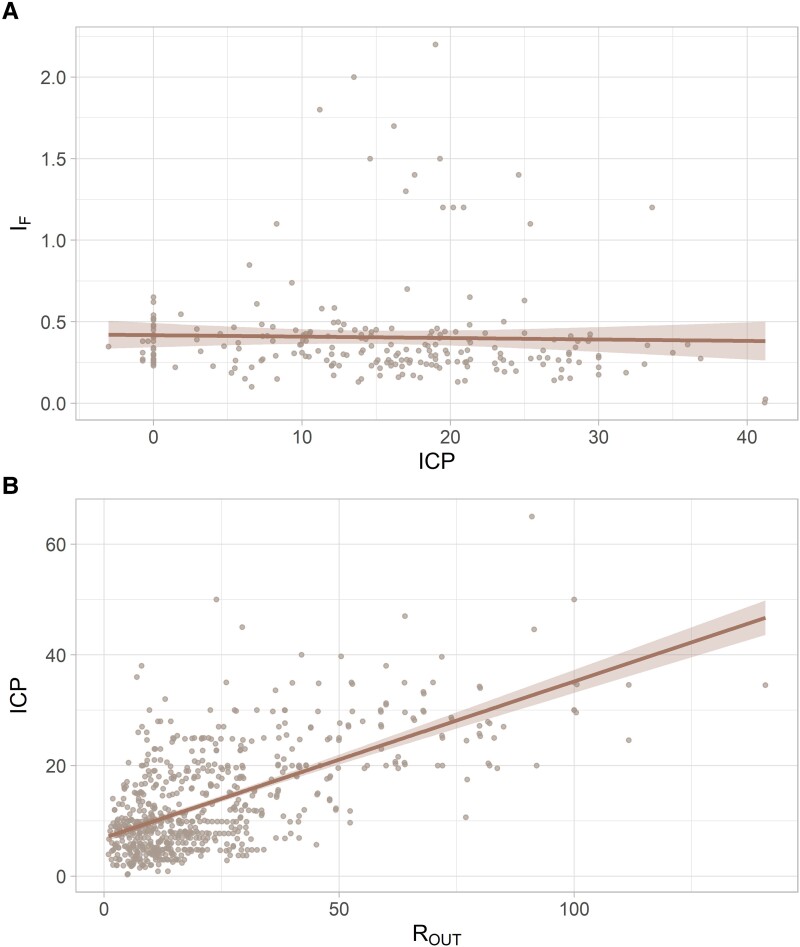
**Relationship between ICP, I_F_ and R_OUT_, over all extracted measurements, including the line of best fit and the 95% confidence interval.** (**A**) ICP versus I_F_ (*N* = 231, *R*² = 0.001, *F* = 0.2, *P* = 0.677). (**B**) ICP and R_OUT_ (*N* = 676, *R*² = 0.405, *F* = 459, *P* < 0.001). Data name: single extracted measurements; data type: continuous; analysis model: linear regression; statistical method: ANOVA; *F*: *F*-value from the ANOVA and *P*: statistical significance of the ANOVA test.

A simple linear regression model was fitted over all extracted data points. As shown in Fig. 3A, there are no changes in I_F_ over the given ICP range (−3 to 40 mmHg), indicating that I_F_ changes independently from ICP (*R*² = 0.0, *P* = 0.68). This relationship is expressed following Eq. (*2*). Conversely, there is a moderate positive correlation between ICP and R_OUT_ (*R*² = 0.41, *P* < 0.001, Fig. 3B). Eq. 3 is used to express this relationship.


(2) $$I_{F}=0\cdot \mathrm{ICP}+0.42$$

(3) $$\mathrm{ICP}=0.28\cdot R_{\mathrm{OUT}}+6.95$$

All relationships extracted from the different analyses (slope and intercept), alongside their *P*-value and *R*² are provided in Table 3.

**Table 3:** Sub-group quantitative synthesis for the relationship between ICP-R_OUT_, and ICP-I_F_

		I_F_			
Group	Sub-groups	Slope [95% CI]	Intercept [95% CI]	*P*-value	*R*²
Whole dataset		0.00 [−0.01, 0.00]	0.42 [0.34, 0.49]	0.677	0.001
Population	Adult	0.00 [−0.01, 0.00]	0.42 [0.33, 0.51]	>0.9	0.000
	Paediatric	−0.01 [−0.01, 0.00]	0.42 [0.36, 0.48]	0.002	0.240
Disease	ABI	−0.01 [−0.02, 0.01]	0.56 [0.34, 0.78]	0.289	0.009
	Brain tumour	−0.01 [−0.02, 0.00]	0.37 [0.33, 0.41]	0.031	0.113
	Neurodegenerative	N/A	N/A	N/A	N/A
	Primary Hydrocephalus	0.00 [−0.02, 0.02]	0.42 [−0.12, 0.97]	0.873	0.004
ICP site	Cranial	0.00 [−0.01, 0.00]	0.42 [0.33, 0.51]	>0.9	0.000
	Lumbar	−0.01 [−0.01, 0.00]	0.42 [0.36, 0.48]	0.002	0.234
SAH versus TBI	SAH	−0.02 [−0.06, 0.01]	1.79 [1.04, 2.53]	0.214	0.101
	TBI	−0.01 [−0.01, 0.00]	0.42 [0.36, 0.49]	<0.001	0.101

		R_OUT_			
Group	Sub-groups	Slope [95% CI]	Intercept [95% CI]	*P*-value	*R*²
Whole dataset		0.28 [0.26, 0.31]	6.95 [6.14, 7.75]	<0.001	0.405
Population	Adult	0.30 [0.27, 0.32]	6.21 [5.33, 7.08]	<0.001	0.439
	Paediatric	0.22 [0.09, 0.34]	11.09 [9.00, 13.19]	<0.001	0.137
Disease	ABI	0.28 [0.23, 0.32]	9.15 [7.44, 10.86]	<0.001	0.487
	Brain tumour	N/A	N/A	N/A	N/A
	Neurodegenerative	0.22 [0.07, 0.36]	5.95 [4.00, 7.89]	0.004	0.125
	Primary Hydrocephalus	0.29 [0.19, 0.38]	12.13 [10.13, 14.13]	<0.001	0.240
ICP Site	Cranial	0.29 [0.27, 0.32]	6.29 [5.39, 6.98]	<0.001	0.455
	Lumbar	0.40 [0.20, 0.60]	12.82 [9.00, 16.65]	<0.001	0.265
SAH versus TBI	SAH	0.26 [0.19, 0.33]	9.90 [6.49, 13.30]	<0.001	0.447
	TBI	0.18 [0.14, 0.23]	10.61 [8.89, 12.33]	<0.001	0.387

### Sub-group analyses

**Figure 4:**
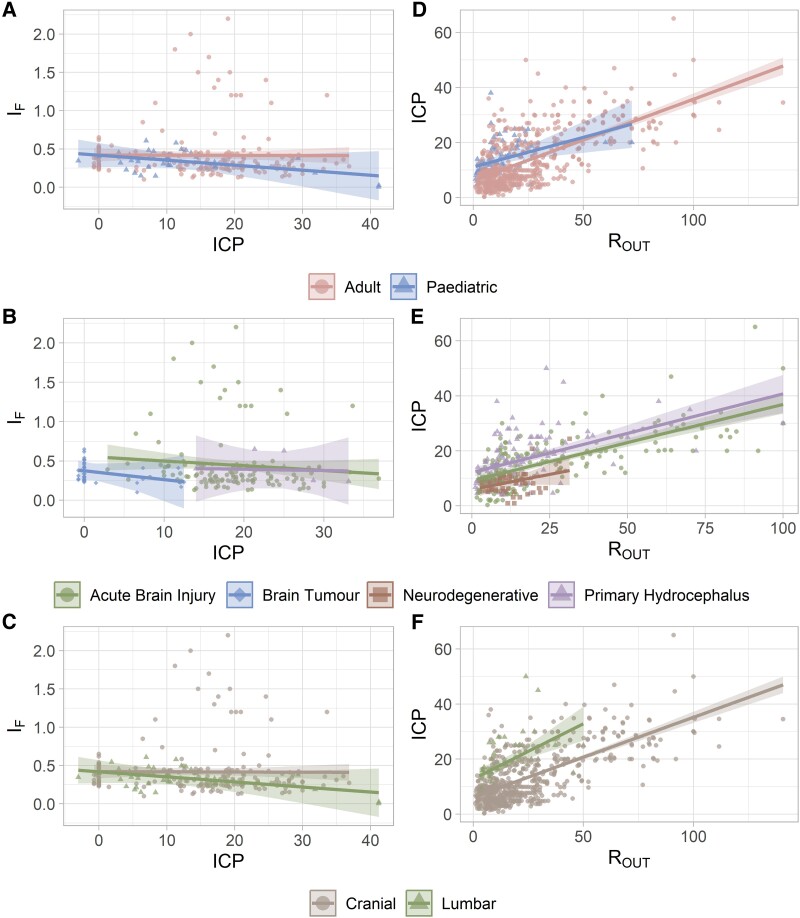
**Relationship between ICP, I_F_ and R_OUT_ for different sub-groups, including the line of best fit and the 95% confidence interval.** ICP versus I_F_ (**A**) per population (adult: *N* = 190, paediatric: *N* = 38; *R*² = 0.015; I_F_: *F* = 0.2, *P* = 0.681; population: *F* = 2, *P* = 0.159; interaction: *F* = 1, *P* = 0.273), (**B**) per disease (ABI: *N* = 129, brain tumour: *N* = 41, primary hydrocephalus: *N* = 9; *R*² = 0.025; I_F_: *F* = 0.0, *P* = 0.826; disease: *F* = 2, *P* = 0.130; interaction: *F* = 0.1, *P* = 0.919) and (**C**) ICP measurement site (cranial: *N* = 191, lumbar: *N* = 40; *R*² = 0.015; I_F_: *F* = 0.2, *P* = 0.676; measurement site: *F* = 2, *P* = 0.156; interaction: *F* = 1, *P* = 0.259). ICP versus R_OUT_ (**D**) per population (adult: *N* = 598, paediatric: *N* = 78; *R*² = 0.423; R_OUT_: *F* = 471, *P* < 0.001; population: *F* = 20, *P* < 0.001; interaction: *F* = 1, *P* = 0.288), (**E**) per disease (ABI: *N* = 151, neurodegenerative: *N* = 66, primary hydrocephalus: *N* = 124; R² = 0.459; R_OUT_: *F* = 238, *P* < 0.001; disease: *F* = 23, *P* < 0.001; interaction: *F* = 0.1, *P* = 0.890), and (**F**) per ICP measurement site (cranial: *N* = 628, lumbar: *N* = 48, *R*² = 0.458; R_OUT_: *F* = 502, *P* < 0.001; measurement site: *F* = 65, *P* < 0.001; interaction: *F* = 2, *P* = 0.211), including the line of best fit and the 95% confidence interval. Data name: single extracted measurements; data type: continuous; analysis model: linear regression; statistical method: ANOVA; *F*: *F*-value from the ANOVA and *P*: statistical significance of the ANOVA test.

Sub-group analysis was conducted by demographic group (adult and paediatric), disease (ABI, brain tumour, neurodegenerative and primary hydrocephalus) and ICP measurement site (cranial and lumbar). Changes in I_F_ have no association with any of the parameters under consideration. Looking at the population (Fig. 4A), the paediatric group reveals a negative relationship between ICP and I_F_ (*R²* = 0.42, *P* = 0.002), whereas these two variables are not intercorrelated in adults (*R²* = 0.00, *P* = 0.93). In terms of diseases, the neurodegenerative group was excluded from the analysis because not enough extracted I_F_ points were available (Supplementary Table 6). Visually, the three remaining disease groups display an overall decrease in I_F_ with increasing ICP (Fig. 4B), but a statistically significant decrease in slope was only reached by the brain tumour group (*R²* = 0.37, *P* = 0.031). The intercept varies across diseases, the highest and lowest being ABI and brain tumour, respectively. Upon closer examination, there is a substantial (*P* < 0.001) difference in I_F_ between SAH and TBI patients, with I_F_ being three times greater for SAH patients (Supplementary Fig. 2A). Considering the ICP measurement site (Fig. 4C), for the lumbar site, ICP is a significant predictor (*R²* = 0.42, *P* = 0.002), with I_F_ decreasing with increasing ICP. For the cranial site, no such correlation could be found.

R_OUT_ remains a strong predictor of ICP (*P* < 0.001) in all the following analyses. The relationship between R_OUT_ and ICP differs considerably across age groups (*R²* = 0.42, *P* < 0.001), with adults having a larger slope but a smaller intercept (Fig. 4D). The brain tumour group was omitted from the analysis due to the insufficient R_OUT_ points (Supplementary Table 6). A substantial difference in the relationship between R_OUT_ and ICP is noted between the remaining disease groups (*R²* = 0.46, *P* < 0.001, Fig. 4E). Specifically, SAH exhibits a similar intercept, but a larger slope compared with the TBI group (Supplementary Fig. 2B). Looking at the ICP measurement site (Fig. 4F), the lumbar site exhibits a significantly larger slope and intercept than the cranial site (*R²* = 0.27, *P* < 0.001).

**Figure 5:**
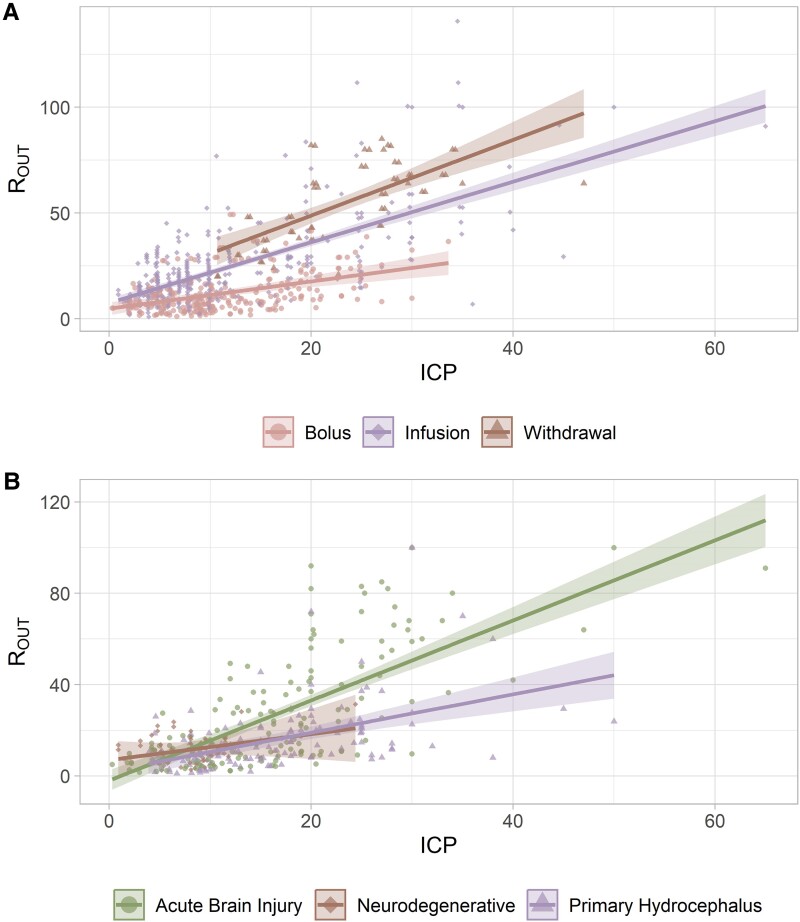
**Relationship between R_OUT_—dependent variable and ICP—independent variable, including the line of best fit and the 95% confidence interval.** (**A**) Per measurement method (bolus: *N* = 261, infusion: *N* = 318, withdrawal: *N* = 59; *R*² = 0.614; ICP: *F* = 742, *P* < 0.001; computation method: *F* = 114, *P* < 0.001; interaction: *F* = 18, *P* < 0.001) and (**B**) per disease (ABI: *N* = 151, neurodegenerative: *N* = 66, primary hydrocephalus: *N* = 124; *R*² = 0.485; ICP: *F* = 250, *P* < 0.001; disease: *F* = 20, *P* < 0.001; interaction: *F* = 13, *P* < 0.001). Data name: single extracted measurements; data type: continuous; analysis model: linear regression; statistical method: ANOVA; *F*: *F*-value from the ANOVA and *P*: statistical significance of the ANOVA test.

The data was also displayed with R_OUT_ being the dependent variable to clarify how ICP may affect R_OUT_. ICP is a strong predictor of R_OUT_ (*P* < 0.001). R_OUT_ is strongly associated with the computation methods (*R²* = 0.61, *P* < 0.001, Fig. 5A), but can only be explained by considering both factors, as the interaction between ICP and computation techniques also shows significant results (*P* < 0.001). The diseases subplot reveals a significant difference (*R²* = 0.49, *P* < 0.001) in R_OUT_ estimations between the ABI, Hydrocephalus and neurodegenerative groups, with a slope for the ABI group twice as high as that of the hydrocephalus group (Fig. 5B). Additionally, there was a significant interaction between ICP and diseases (*P* < 0.001).

Lastly, we compared estimated versus measured I_F_. Median measured I_F_ differed significantly from the estimated I_F_ derived from Davson’s equation, except for the primary hydrocephalus group (*P* = 0.154, Supplementary Table 7).

### Secondary analysis

Initially, a multiple regression model was constructed including ICP, population type, disease, and ICP measurement site as predictors of I_F_. No predictor emerged as significant, as was predicted based on earlier analysis (*R²* = 0.04, *P* = 0.059). The effect of the Study ID was explored through a mixed effect model by incorporating it as a random intercept effect alongside the previously mentioned fixed effects. The effect of Study ID on intercept was meaningful (*P* < 0.001). Incorporating the Study ID as a random effect made ICP a predictor of I_F_ (*R²m* = 0.22, *R²c* = 0.91, *P* < 0.001). While this effect was statistically significant, it is likely clinically minimally meaningful with a negative slope of −0.01.

Likewise, a second multiple regression model was built including R_OUT_, population type, disease, and ICP measurement site as predictors of ICP. Every predictor was found to be significant (*R²* = 0.55, *P* < 0.001) for estimating ICP. Using a mixed effect model, the Study ID's impact was once more investigated by adding it as a random intercept effect. The random intercept changed significantly (*R²m* = 0.43, *R²c* = 0.67, *P* < 0.001) comparing the two models. On the other hand, population type and ICP measurement site were insignificant with the added random effect of Study ID. Only R_OUT_ (*P* < 0.001) and disease type (*P* = 0.009) were retained.

## Discussion

### Interpretation of the results

This is the first systematic review that examines the relationship between ICP, I_F_ and R_OUT_. Based on the compilation of the data from all the investigations, I_F_ is constant over the range of ICP reported, confirming the assumptions made in the literature.^36-38^ On the other hand, ICP is strongly positively correlated with R_OUT_.

For the vast majority of sub-groups, I_F_ is predicted to be about 0.42 mL/min, which is greater than the constant production of roughly 0.33 mL/min reported in the literature for healthy individuals.^39^ CSF production rate was shown to vary substantially amongst diseases.^40,41^ Patients with ABI (SAH and intracranial haemorrhage) have the highest production rate (2–3 mL/min), followed by those with hydrocephalus (1.37–1.57 mL/min) and brain tumours (0.43–0.6 mL/min). Neurodegenerative diseases, particularly Alzheimer's disease, displayed the lowest production rates (0.20 mL/min).^42^ A linear relationship between R_OUT_ and ICP was found. The estimated CSF production rate was 0.28 mL/min, and the estimated sagittal sinus pressure was 6.95 mmHg, which is equivalent to studies that evaluated P_ss_ through invasive means. The various sub-group studies provided a deeper understanding of these associations.

As reported in the literature,^43,44^ a lower I_F_ and a greater P_SS_ have been identified in the paediatric population with hydrocephalus when compared with the adult population. These differences were validated in the collected data when evaluating the association of ICP and R_OUT_, with I_F_ and P_SS_ estimated as 0.22 mL/min and 11.1 mmHg, respectively, in the paediatric population, and 0.30 mL/min and 6.21 mmHg in the adults. There is a significant difference among the disease groups when it comes to the estimated P_SS_, which is 12.13 mmHg for Primary Hydrocephalus, 9.15 mmHg for ABIs and 5.95 mmHg for Neurodegenerative. P_SS_ increases in cases of chronic hydrocephalus from the collapse of the sagittal sinus;^45^ in obese patients with IIH, it tends to increase due to the collapse of sinuses below the torcular.^46^ Both mechanisms result in a decrease in CSF absorption through the arachnoid granulations and an elevation in ICP. Based on the pathophysiology of neurodegeneration, it is possible that the decrease in P_SS_ occurs as a consequence of the decreasing brain volume allowing for improved venous drainage and possibly decreased pressure. The results indicate that, in comparison to cranial ICP, lumbar ICP estimation displays a considerably larger slope and intercept. The reason for this phenomenon can be attributed to the sensitivity of ICP to the patient’s body position during the process with ICP readings being higher for lumbar measurements compared with the cranial measurements.^47^ Furthermore, the discrepancy in estimated P_SS_ (12.83 mmHg for the lumbar site; 6.19 mmHg for the cranial site) can be explained by a possible increase in P_SS_ during infusion tests conducted in the lumbar area where patients are typically in the lateral recumbent position (versus supine)^48^ due to reduced venous drainage.

For a given ICP, constant rate withdrawal yields the highest estimation of R_OUT_, followed by infusion, and bolus.^49-51^ During infusion tests, an increase in ICP is likely to provoke an autoregulatory response resulting in vasodilatation, in turn causing additional ICP increases potentially leading to overestimation of R_OUT_. This effect is likely not pronounced during bolus tests, where the change in ICP is transient and likely will not cause a strong autoregulatory response. Nevertheless, Kosteljanetz^51^ showed a strong correlation between R_OUT_ calculated using the two approaches, which couldn’t be verified in our dataset due to a lack of dual measurements in the same patients.

Increases in brain or cerebral blood volume may occur after ABIs, leading to a cascade of compensatory mechanisms described by the Monro–Kellie doctrine. This theory refers to the constant sum of the cranium contents—brain parenchyma, CSF and intracranial blood—as the brain is contained in a rigid enclosing: the skull. Through compensating mechanisms, a rise in one of these volumes results in a drop in either or both others. Theoretically, this concept has significant consequences for both decreased CSF volume and elevated intracranial pressure,^52^ particularly relevant in ABIs. An initial decrease in cerebral blood volume through the compression of the larger cerebral veins and increased drainage is followed by a decrease in CSF volume via shifting the CSF into the spinal canal. We can only speculate at which point or why there is an increase in R_OUT_ in ABI but we must note that R_OUT_ represents not only the reabsorption through the arachnoid granulations but the whole pathway of CSF circulation from the production site, which is possibly influenced by the oedema. This hypothesis is supported by our analysis, suggesting that ABI patients have a higher increase in R_OUT_ with increasing ICP compared with other disease groups.

Particular attention was paid to the association between I_F_, R_OUT_ and ICP in TBI and SAH. In comparison to TBI, SAH revealed a higher rate of I_F_. This observation can also be found in the literature^53^ and may explain the emergence of post-SAH hydrocephalus from methods other than the initial haemorrhage. Considering the association between ICP and R_OUT_, the difference between TBI and SAH is marginal.

Davson’s equation can be disputed by comparing the I_F_ estimations (based on Davson’s equation) against the I_F_ measurements (measured invasively) for each sub-group. The values did not differ for the hydrocephalus group but were different for patients with ABI. The suitability for CSF-related disorders might be owed to Davson’s equation being based on data from healthy subjects and animal experiments with relatively constant brain and blood volumes. Contrarily, the significant difference for other pathologies highlights the need for including additional components (i.e. accounting for further mechanisms that affect ICP).

The secondary analysis explored the effect of each study protocol and centre-specific differences on the obtained results. There was a significant impact of Study ID on the derived models. Whereas R_OUT_ and the disease group remained highly significant predictors of ICP, the other variables lost their significance. In contrast, when the variance resulting from different studies was considered as a random effect predicting I_F_, ICP emerged as a significant but clinically likely negligible predictor of I_F_.

### Limitations

There are several limitations pertaining to the data analysis and the quality of the underlying literature. Research differed widely in terms of sample size, patient age, illness type and severity, treatment environment, and study design. This heterogeneity was reflected in the high *I*² values, indicating that the pooled estimates should be interpreted cautiously. To address this variability, sub-group analyses were conducted. Although the dataset presented in this study is subject to potential confounding factors, these biases were mitigated through the use of multivariable analyses, enhancing the validity and reliability of the findings. The literature is dominated by studies from a narrow range of research groups, with studies mainly conducted prior to the year 2000. Few study groups have focused on this subject; therefore, despite all precautions, some patients may appear twice or more across different studies. Only one measurement per patient was provided, which increases the uncertainty around the results.

Due to the already complex nature of this study, only two groups were formed to examine the age dependence of I_F_ and R_OUT_ (i.e. below versus above 16). The CSF formation rate is highest in young adults and diminishes with age, reaching approximately 50% of its initial value by the age of 70.^54^ Additionally, R_OUT_ linearly depends on age.^55^ Cerebral blood flow and pressure descriptors were not presented alongside the I_F_ measurements, which poses a significant constraint to the interpretation of the data. Although the correlation between blood flow and CSF production rate in dogs has been demonstrated,^56^ investigations in humans are still required. Further limitations are associated with employing Davson’s equation to analyse the results. In this equation, ICP is only described by the CSF compartment parameters. It completely disregards the impact of changes in brain tissue volume and rapid vascular mechanisms. Strong correlations between ICP and P_SS_ have been demonstrated in IIH,^8^ showing the need for a modified Davson’s equation that takes such a relationship into account.

Davson *et al*.^57^ described I_F_ as originating from the choroid plexus. However, numerous studies have identified potential non-choroid plexus sites of CSF production, such as the brain, cerebral superficial subarachnoid space, perivascular system, and spinal cord, along with alternative production mechanisms. The premise of Davson’s equation assumes that all produced CSF is absorbed into the venous sinuses via the arachnoid granulations. Nevertheless, neonates and infants have a limited number of arachnoid granulations, suggesting a developmental progression of these structures as they age.^58,59^ Other CSF absorption sites, beyond the arachnoid granulations, have also been reported—including the perineural olfactory sheath, retro-orbital tissue, inner ear and spinal canal—challenging the assumption proposed by Davson.^1^ Additionally, Ligocki *et al*.^60^ have demonstrated a continuous and contiguous CSF flow extending from the central nervous system to the peripheral nervous system, suggesting additional mechanisms for CSF absorption. Furthermore, Hassin^61^ explored the role of the interstitial fluid in CSF physiology, introducing another possible pathway for CSF circulation.

Lastly, while I_F_ represents an actual measurement, R_OUT_ is an estimation based on a defined set of formulas, derived from the result of a change in CSF volume and Davson’s equation itself, and thus it may not reflect true R_OUT_, as exemplified by the differences seen when using different computational methods.

## Conclusion

R_OUT_ and I_F_ have been studied in various acute and chronic diseases across different age groups using different methods and interventions. I_F_ remains relatively constant irrespective of ICP. Conversely, the association between ICP and R_OUT_ is not constant, as expected. This relationship depends on different factors including age, type of disease and ICP measurement site and does not seem to be readily explained by Davson’s equation alone. These results imply that the equation cannot be applied without restrictions. The addition of other factors affecting the cerebrovascular system will ultimately allow for more accurate estimations.

## Supplementary Material

fcaf044_Supplementary_Data

## Data Availability

The extracted data is available from the corresponding author upon reasonable request. The codes are available in the Supplementary materials.
