# Supplementary material for: CSF production rate, resistance to reabsorption, and intracranial pressure: a systematic review and meta-analysis
Source: Brain Commun. 2025 Jan 30;7(1):fcaf044. doi: 10.1093/braincomms/fcaf044 (PMC11822472; doi:10.1093/braincomms/fcaf044)
Supplement: fcaf044_Supplementary_Data [file fcaf044_supplementary_data.pdf]

Table 1. PRISMA Checklist

Section and Topic	Item #	Checklist item	Location where item is reported
**TITLE**			
Title	1	Identify the report as a systematic review.	1
**ABSTRACT**			
Abstract	2	See the PRISMA 2020 for Abstracts checklist.	2
**INTRODUCTION**			
Rationale	3	Describe the rationale for the review in the context of existing knowledge.	7
Objectives	4	Provide an explicit statement of the objective(s) or question(s) the review addresses.	7
**METHODS**			
Eligibility criteria	5	Specify the inclusion and exclusion criteria for the review and how studies were grouped for the syntheses.	8
Information sources	6	Specify all databases, registers, websites, organisations, reference lists and other sources searched or consulted to identify studies. Specify the date when each source was last searched or consulted.	8
Search strategy	7	Present the full search strategies for all databases, registers and websites, including any filters and limits used.	8
Selection process	8	Specify the methods used to decide whether a study met the inclusion criteria of the review, including how many reviewers screened each record and each report retrieved, whether they worked independently, and if applicable, details of automation tools used in the process.	8-9
Data collection process	9	Specify the methods used to collect data from reports, including how many reviewers collected data from each report, whether they worked independently, any processes for obtaining or confirming data from study investigators, and if applicable, details of automation tools used in the process.	9
Data items	10a	List and define all outcomes for which data were sought. Specify whether all results that were compatible with each outcome domain in each study were sought (e.g. for all measures, time points, analyses), and if not, the methods used to decide which results to collect.	9
	10b	List and define all other variables for which data were sought (e.g. participant and intervention characteristics, funding sources). Describe any assumptions made about any missing or unclear information.	9
Study risk of bias assessment	11	Specify the methods used to assess risk of bias in the included studies, including details of the tool(s) used, how many reviewers assessed each study and whether they worked independently, and if applicable, details of automation tools used in the process.	9
Effect measures	12	Specify for each outcome the effect measure(s) (e.g. risk ratio, mean difference) used in the synthesis or presentation of results.	9-10
Synthesis methods	13a	Describe the processes used to decide which studies were eligible for each synthesis (e.g. tabulating the study intervention characteristics and comparing against the planned groups for each synthesis (item #5)).	8
	13b	Describe any methods required to prepare the data for presentation or synthesis, such as handling of missing summary statistics, or data conversions.	9
	13c	Describe any methods used to tabulate or visually display results of individual studies and syntheses.	9
	13d	Describe any methods used to synthesize results and provide a rationale for the choice(s). If meta-analysis was performed, describe the model(s), method(s) to identify the presence and extent of statistical heterogeneity, and software package(s) used.	10
	13e	Describe any methods used to explore possible causes of heterogeneity among study results (e.g. subgroup analysis, meta-regression).	9-10
	13f	Describe any sensitivity analyses conducted to assess robustness of the synthesized results.	9-10
Reporting bias assessment	14	Describe any methods used to assess risk of bias due to missing results in a synthesis (arising from reporting biases).	NA
Certainty assessment	15	Describe any methods used to assess certainty (or confidence) in the body of evidence for an outcome.	NA

Section and Topic	Item #	Checklist item	Location where item is reported
**RESULTS**			
Study selection	16a	Describe the results of the search and selection process, from the number of records identified in the search to the number of studies included in the review, ideally using a flow diagram.	11
	16b	Cite studies that might appear to meet the inclusion criteria, but which were excluded, and explain why they were excluded.	NA
Study characteristics	17	Cite each included study and present its characteristics.	11
Risk of bias in studies	18	Present assessments of risk of bias for each included study.	11
Results of individual studies	19	For all outcomes, present, for each study: (a) summary statistics for each group (where appropriate) and (b) an effect estimate and its precision (e.g. confidence/credible interval), ideally using structured tables or plots.	11
Results of syntheses	20a	For each synthesis, briefly summarise the characteristics and risk of bias among contributing studies.	11
	20b	Present results of all statistical syntheses conducted. If meta-analysis was done, present for each the summary estimate and its precision (e.g. confidence/credible interval) and measures of statistical heterogeneity. If comparing groups, describe the direction of the effect.	11-14
	20c	Present results of all investigations of possible causes of heterogeneity among study results.	14
	20d	Present results of all sensitivity analyses conducted to assess the robustness of the synthesized results.	14
Reporting biases	21	Present assessments of risk of bias due to missing results (arising from reporting biases) for each synthesis assessed.	NA
Certainty of evidence	22	Present assessments of certainty (or confidence) in the body of evidence for each outcome assessed.	NA
**DISCUSSION**			
Discussion	23a	Provide a general interpretation of the results in the context of other evidence.	15-18
	23b	Discuss any limitations of the evidence included in the review.	18-19
	23c	Discuss any limitations of the review processes used.	18-19
	23d	Discuss implications of the results for practice, policy, and future research.	19
**OTHER INFORMATION**			
Registration and protocol	24a	Provide registration information for the review, including register name and registration number, or state that the review was not registered.	8
	24b	Indicate where the review protocol can be accessed, or state that a protocol was not prepared.	8
	24c	Describe and explain any amendments to information provided at registration or in the protocol.	NA
Support	25	Describe sources of financial or non-financial support for the review, and the role of the funders or sponsors in the review.	31
Competing interests	26	Declare any competing interests of review authors.	31
Availability of data, code and other materials	27	Report which of the following are publicly available and where they can be found: template data collection forms; data extracted from included studies; data used for all analyses; analytic code; any other materials used in the review.	31

Table 2. Characteristics of 25 studies included in the meta-analysis. *AS:* Aqueduct Stenosis, *AVM:* Arteriovenous Malformation, *BT:* Brain Tumour, *F:* Female, *HPH:* High-Pressure Hydrocephalus, *IIH:* Idiopathic Intracranial Hypertension, *M:* Male, *NPH:* Normal-Pressure Hydrocephalus, *NR:* Not Reported, *SAH:* Subarachnoid Haemorrhage, *TBI:* Traumatic Brain Injury

Ref	Author (Year)	Results Format	Variable	Population	Sex	Number of Patients	Disease	Patients Specifics
1	Alperin, N. (2016)	Table	I_F	Adults	F	7	IIH	
2	Borgesen, S. E. (1987)	Graph, Regression Line	R_{OUT}	Adults	NR	230	HPH, IIH, NPH	- 168 NPH + 23 IIH + 39 HPH
3	Cutler, R. W. P. (1968)	Graph, Regression Line	I_F	Paediatric	F, M	12	BT, Cerebral Infection	- 4 Females, 8 Males<br>- 8 Cerebral Infection, 4 BT
4	Eide, P. K. (2001)	Graph, Table	R_{OUT}	Paediatric	F, M	28	Craniosynostosis, Hydrocephalus	6 Females, 22 Males
5	Eide, P. K. (2003)	Graph, Regression Line	R_{OUT}	Adults	F, M	16	NPH	11 Females, 6 Males
6	Gideon, P. (1994)	Table	R_{OUT}	Adults, Paediatric	F, M	12	IIH	- 8 Females, 4 Males<br>- 1 Paediatric, 11 Adults
7	Gjerris, F. (1985)	Table	R_{OUT}	Adults, Paediatric	F, M	14	IIH	- 10 Females, 4 Males<br>- 2 Paediatrics, 12 Adults
8	Gjerris, F. (1987)	Table	R_{OUT}	Adults	F, M	11	HPH, SAH	9 Females, 2 Males
9	Hansen, K. (1987)	Table	R_{OUT}	Adults	F, M	4	Meningitis, Spinal Tumour	- 3 Females, 1 Male<br>- 3 Spinal Tumour, 1 Meningitis
10	Hayashi, M. (1982)	Table	I_F	Adults	F, M	9	AS, BT, Chronic SDH, IIH, NPH	- 4 Females, 5 Males<br>- 3 BT, 1 AS, 1 Chronic SDH, 2 IIH, 2 NPH
11	Hayashi, M. (1991)	Table	I_F, R_{OUT}	Adults	F, M	94	BT, Hydrocephalus, Intracranial Haemorrhage, IIH, Meningitis, SAH, Superior Sagittal Sinus Thrombosis	- 46 Females, 48 Males<br>- 17 w/o plateau wave, 77 with plateau wave
12	Janny, P. (1981)	Table	R_{OUT}	Adults, Paediatric	F, M	22	AVM, IIH, Meningioma, Meningitis	- 10 Females, 12 Males<br>- 8 Paediatrics, 14 Adults<br>- 1 AVM, 1 Meningioma, 4 Meningitis, 16 IIH

13	Kosteljanetz, M. (1984)	Equation, Graph, Regression Line, Table	R_{OUT}	Adults	F, M	17	SAH	12 Females, 5 Males
14	Kosteljanetz, M. (1986)	Graph, Regression Line	R_{OUT}	Adults	NR	62	Hydrocephalus, SAH, TBI	17 SAH, 30 Hydrocephalus, 15 TBI
15	Kosteljanetz, M. (1986)	Graph, Regression Line, Table	R_{OUT}	Adults	F, M	16	TBI	5 Females, 11 Males
16	Kosteljanetz, M. (1986)	Graph, Regression Line, Table	R_{OUT}	Adults	F, M	26	NPH	14 Females, 12 Males
17	Lorenzo, A. V. (1970)	Graph, Regression Line	I_F	Adults, Paediatric	F, M	12	AS, Cerebral Infection, Hydrocephalus, Meningitis, NPH	- 3 Females, 9 Males<br>- 10 Paediatrics, 2 Adults
18	Marmarou, A. (1987)	Graph, Regression Line, Table	I_F, R_{OUT}	Adults	NR	28	TBI	- 26 for I_F measurements before prior to withdrawal of CSF<br>- 28 for I_F + R_{OUT} measurements
19	Marmarou, A. (1996)	Graph, Regression Line	R_{OUT}	Adults	NR	75	Atrophy, HPH, IIH, NPH, TBI	31 TBI, 11 IIH/TBI, 18 Atrophy/TBI, 7 NPH/TBI, 8 HPH/TBI
20	Rubin, R. C. (1966)	Table	I_F	Adults	NR	11	BT	
21	Sahuquillo, J. (1991)	Table	R_{OUT}	Adults	F, M	54	Hydrocephalus	- 24 Females, 30 Males<br>- 8 Active Hydrocephalus, 46 Compensated Hydrocephalus (16 stable + 30 unstable)
22	Shapiro, K. (1985)	Table	R_{OUT}	Paediatric	NR	13	Hydrocephalus	
23	Shapiro, K. (1986)	Graph, Regression Line	R_{OUT}	Paediatric	NR	20	Hydrocephalus	
24	Silverberg, G. D. (2002)	Values	I_F	Adults, Paediatric	NR	30	Hydrocephalus, Parkinson	14 Parkinson, 6 Acute Hydrocephalus, 10 Chronic Hydrocephalus
25	Stocchetti, N. (1994)	Table	I_F, R_{OUT}	Adults	F, M	17	SAH	4 Females, 13 Males

Table 3. Methodological characteristics of included studies. EVD: Extraventricular Drain

Ref	Author (Year)	Intervention	ICP Measurement Site	Range of ICP	Range of I_F (mL/min)	Range of R_{OUT} (mmHg/mL/min)	Methods I_F/R_{OUT}
1	Alperin, N. (2016)	Withdrawal	Lumbar Puncture	25 – 40 cmH_2O	0.24 - 0.65		1
2	Borgesen, S. E. (1987)	Infusion	EVD	2 – 45 mmHg		1 – 141	4
3	Cutler, R. W. P. (1968)	Infusion	Lumbar Puncture	0 – 200 mmCSF	0.2 - 0.6		2
4	Eide, P. K. (2001)	Infusion	Wire	3.9 – 22.6 mmHg		5.6 – 26	7
5	Eide, P. K. (2003)	Infusion	EVD	0.9 – 11.5 mmHg		8.7 – 23.7	7
6	Gideon, P. (1994)	Infusion	Lumbar Puncture	18 – 50 mmHg		7.3 – 37.2	Not provided
7	Gjerris, F. (1985)	Infusion	Lumbar Puncture	8 – 45 mmHg		14.29 – 50	4
8	Gjerris, F. (1987)	Infusion	EVD	18 – 65 mmHg		29 – 100	4
9	Hansen, K. (1987)	Infusion	EVD, Wire	30 – 35 mmHg		38.46 – 100	4
10	Hayashi, M. (1982)	Withdrawal	EVD	12 – 35 mmHg	0.25 - 0.31		1
11	Hayashi, M. (1991)	Infusion	EVD	27 – 36 mmHg	0.36 - 0.39	7 – 24.4	1, 4
12	Janny, P. (1981)	Infusion	EVD	13 – 38 mmHg		8 – 100	Not provided
13	Kosteljanetz, M. (1984)	Bolus, Infusion, Withdrawal	EVD	4.2 – 34 mmHg		11.5 – 85	5, 7, 8
14	Kosteljanetz, M. (1986)	Bolus, Infusion, Withdrawal	EVD	0 – 35 mmHg		1.97 – 81.73	5, 7, 8
15	Kosteljanetz, M. (1986)	Bolus, Withdrawal	EVD	0.3 – 47 mmHg		1.6 – 64	5, 8
16	Kosteljanetz, M. (1986)	Bolus	EVD	5 – 24.4 mmHg		2.5 – 31.4	5
17	Lorenzo, A. V. (1970)	Infusion	Lumbar Puncture	25 – 561 mmCSF	0 - 0.55		2
18	Marmarou, A. (1987)	Bolus, Withdrawal	EVD	3 – 38 mmHg	0.13 - 0.85	2.25 – 35.62	3, 5
19	Marmarou, A. (1996)	Bolus	Lumbar Puncture	7.64 – 17 mmHg		2.77 – 11.11	6
20	Rubin, R. C. (1966)	Infusion	EVD	-10 – 170 mmH_2O	0.1 - 0.54		2
21	Sahuquillo, J. (1991)	Bolus	Wire	8.1 – 25.5 mmHg		14.3 – 38.8	5
22	Shapiro, K. (1985)	Bolus	EVD	4.5 – 27 mmHg		1.7 – 18.6	6
23	Shapiro, K. (1986)	Bolus	EVD	4.5 – 25 mmHg		1 – 25	6
24	Silverberg, G. D. (2002)	Withdrawal	EVD	14 – 16 cmH_2O	0.25 – 0.42		1
25	Stocchetti, N. (1994)	Bolus, Withdrawal	EVD	8.3 – 33.6 mmHg	0.5 – 1.8	4.7 – 36.5	3, 5

Table 4. Quality Assessment of Included Studies, following the NIH Quality Assessment Tool for Case Series Studies.
NR: Not Reported, NA: Not Assessed

Author (Year)Reference	NIH Quality Assessment Tool for Case Series Studies Criteria Met									Quality (Total)
	Q1	Q2	Q3	Q4	Q5	Q6	Q7	Q8	Q9	
Alperin, N. (2016)[1]	Yes	Yes	NR	Yes	Yes	Yes	NR	Yes	Yes	**Good (7)**
Borgesen, S. E. (1987)[2]	Yes	Yes	NR	No	No	Yes	NR	Yes	Yes	**Fair (5)**
Cutler, R. W. P. (1968)[3]	Yes	Yes	NR	Yes	Yes	Yes	NR	Yes	Yes	**Good (7)**
Eide, P. K. (2001)[4]	Yes	Yes	NR	Yes	Yes	Yes	NR	Yes	Yes	**Good (7)**
Eide, P. K. (2003)[5]	Yes	Yes	Yes	Yes	Yes	Yes	NR	Yes	Yes	**Good (7)**
Gideon, P. (1994)[6]	Yes	Yes	NR	No	Yes	Yes	NR	Yes	Yes	**Fair (6)**
Gjerris, F. (1985)[7]	Yes	Yes	Yes	No	Yes	Yes	Yes	NA	Yes	**Good (7)**
Gjerris, F. (1987)[8]	Yes	Yes	Yes	Yes	Yes	Yes	Yes	Yes	Yes	**Good (9)**
Hansen, K. (1987)[9]	Yes	Yes	NR	Yes	No	No	Yes	NA	Yes	**Fair (5)**
Hayashi, M. (1982)[10]	Yes	Yes	NR	Yes	Yes	Yes	NR	NA	Yes	**Fair (6)**
Hayashi, M. (1991)[11]	Yes	Yes	NR	Yes	Yes	Yes	NR	No	No	**Fair (5)**
Janny, P. (1981)[12]	Yes	Yes	Yes	No	No	Yes	NR	NA	Yes	**Fair (5)**
Kosteljanetz, M. (1984)[13]	Yes	Yes	Yes	Yes	Yes	No	Yes	No	Yes	**Good (7)**
Kosteljanetz, M. (1986)[14]	Yes	No	NR	No	No	No	NR	No	Yes	**Poor (2)**
Kosteljanetz, M. (1986)[15]	Yes	Yes	No	Yes	No	Yes	Yes	Yes	Yes	**Good (7)**
Kosteljanetz, M. (1986)[16]	Yes	Yes	Yes	Yes	Yes	Yes	NR	Yes	Yes	**Good (8)**
Lorenzo, A. V. (1970)[17]	Yes	Yes	NR	No	Yes	Yes	NR	NA	Yes	**Fair (5)**
Marmarou, A. (1987)[18]	Yes	No	Yes	Yes	Yes	Yes	NR	No	Yes	**Fair (6)**
Marmarou, A. (1996)[19]	Yes	Yes	Yes	Yes	Yes	Yes	Yes	Yes	Yes	**Good (9)**
Rubin, R. C. (1966)[20]	Yes	Yes	NR	No	Yes	No	NR	NA	Yes	**Fair (4)**
Sahuquillo, J. (1991)[21]	Yes	Yes	Yes	Yes	Yes	Yes	Yes	Yes	No	**Good (8)**
Shapiro, K. (1985)[22]	Yes	Yes	Yes	Yes	Yes	Yes	NR	Yes	Yes	**Good (8)**
Shapiro, K. (1986)[23]	Yes	Yes	NR	Yes	Yes	Yes	NR	No	Yes	**Fair (6)**
Silverberg, G. D. (2002)[24]	Yes	No	NR	No	Yes	Yes	Yes	Yes	No	**Fair (5)**
Stocchetti, N. (1994)[25]	Yes	Yes	NR	Yes	Yes	Yes	Yes	Yes	Yes	**Good (8)**

Table 5. Formula Table

Number	Formula	Acquisition Method
1	$I_F = \frac{\Delta CSF_{volume}}{t}$	Quotient of the difference of volume pre- and post- CSF withdrawal (ΔCSF_{volume}), and the time elapsed during withdrawal (t).
2	$I_F = V_i \cdot \frac{C_i - C_o}{C_o}$	Determined following the infusion of an artificial CSF (of volume V_i) containing a tracer (of concentration C_i), by measuring the tracer's dilution (from the output concentration C_o). (Heisey, 1962)
3	$I_F = \frac{PVI_W}{t_1} \cdot log\left(\frac{P_1}{P_m}\right)$ $with\ PVI_W = \frac{\Delta V}{log\left(\frac{P_0}{P_m}\right)}$	Estimation based on the pressure-volume index from withdrawal (PVI_W) – computed from the volume of CSF bolus withdrawal (ΔV), baseline pressure (P_0) and minimal pressure developed after withdrawal (P_m) – a measurement of pressure (P_1) at a certain time on the return trajectory (t_1), and P_m. (Marmarou, 1987)
4	$R_{OUT} = Slope\ between\ ICP\ and\ V_{abs}$ $V_{abs} = V_{in} + V_{fr} - V_{out}$	Conductance to CSF outflow reported as the slope of the linear relationship linking the CSF volume absorbed (V_{abs}) and ICP. Resistance to CSF outflow corresponds to the inverse of the conductance. (Boergsen, 1978)
5	$R_{OUT} = \frac{t \cdot P_0}{PVI_i \cdot log\left[\frac{P_t}{P_p} \cdot \left(\frac{P_p - P_0}{P_t - P_0}\right)\right]}$ $with\ PVI_i = \frac{\Delta V}{log\left(\frac{P_p}{P_m}\right)}$	Estimation based on the pressure-volume index from injection (PVI_i) – computed from the volume of CSF bolus injection (ΔV), baseline pressure (P_0) and peak pressure developed after injection (P_p) – a measurement of pressure (P_t) at a certain time on the return trajectory (t), and P_p. (Marmarou, 1975)
6	$R_{OUT} = \frac{P_0}{PVI_i \cdot log\left[\frac{P_t}{P_p} \cdot \left(\frac{P_p - P_0}{P_t - P_0}\right)\right]}$	Readaptation of (Marmarou, 1975) formula, using the same parameters.
7	$R_{OUT} = \frac{P_{eq} - P_{init}}{\Delta I}$	Quotient of the difference of equilibrium pressure pre-infusion (P_{init}) and post-infusion (P_{eq}), and the constant infusion rate (ΔI). (Kosteljanetz, 1984)
8	$R_{OUT} = \frac{P_{eq} - P_{init}}{-\Delta I}$	Quotient of the difference of equilibrium pressure pre-withdrawal (P_{init}) and post-withdrawal (P_{eq}), and the constant withdrawal rate ($-\Delta I$). (Kosteljanetz, 1984)

Table 6. Specifics about the number of extracted points for each category

**Variable**	**Sub-groups**	**R_{OUT} extracted points**	**I_F extracted points**
Whole dataset		676	231
Population	Adult	598	190
	Paediatric	78	38
	Mixed	0	3
Disease	ABI	151	129
	Brain Tumour	5	41
	Neurodegenerative	66	1
	Primary Hydrocephalus	124	9
	Mixed	330	51
ICP Site	Cranial	628	191
	Lumbar	48	40
R_{OUT} Computation Method	Bolus	261	
	Infusion	318	
	Withdrawal	59	
	Unknown	38	

Table 7. Comparison between measured I_F and Davson's estimated I_F for subgroups having both information

Variable	Sub-groups	Median	Wilcoxon test
Whole dataset		0.32	< 0.001
Population	Adult	0.31	< 0.001
	Paediatric	0.36	< 0.001
Disease	ABI	0.31	< 0.001
	Primary Hydrocephalus	0.4	0.154
TBI vs SAH	SAH	1.3	< 0.001
	TBI	0.29	< 0.001
ICP Site	Cranial	0.31	< 0.001
	Lumbar	0.36	0.0204

Figure 1. Full Search Strategy

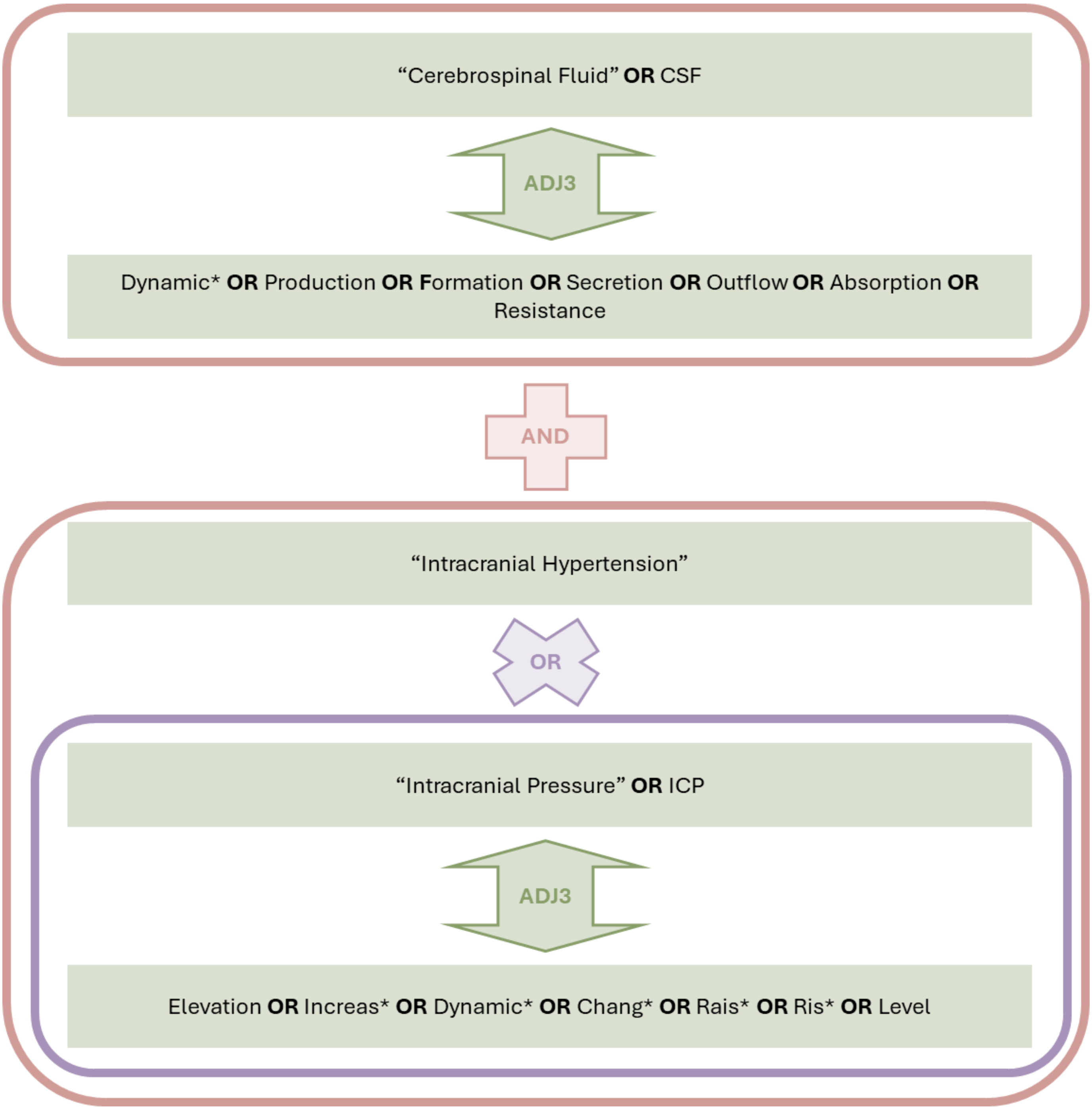

Figure 2. Relationship between intracranial pressure (ICP), CSF production rate (I_F) and resistance to outflow (R_{OUT}), for subarachnoid haemorrhage (SAH) and traumatic brain injury (TBI), including the line of best fit and the 95% confidence interval. (A) ICP vs. I_F (SAH: N = 17, TBI: N = 112; R^2 = 0.807; I_F: F = 6, *P* = 0.018; Disease: F = 513, *P* < 0.001; Interaction: F = 4, *P* = 0.037). (B) ICP vs. R_{OUT} (SAH: N = 70, TBI: N = 96; R^2 = 0.468; R_{OUT}: F = 136, *P* < 0.001; Disease: F = 3, *P* = 0.110; Interaction: F = 4, *P* = 0.062). Data Name: Single extracted measurements; Data type: Continuous; Analysis model: Linear Regression; Statistical method: ANOVA; F: F-value from the ANOVA; *P*: Statistical significance of the ANOVA test.

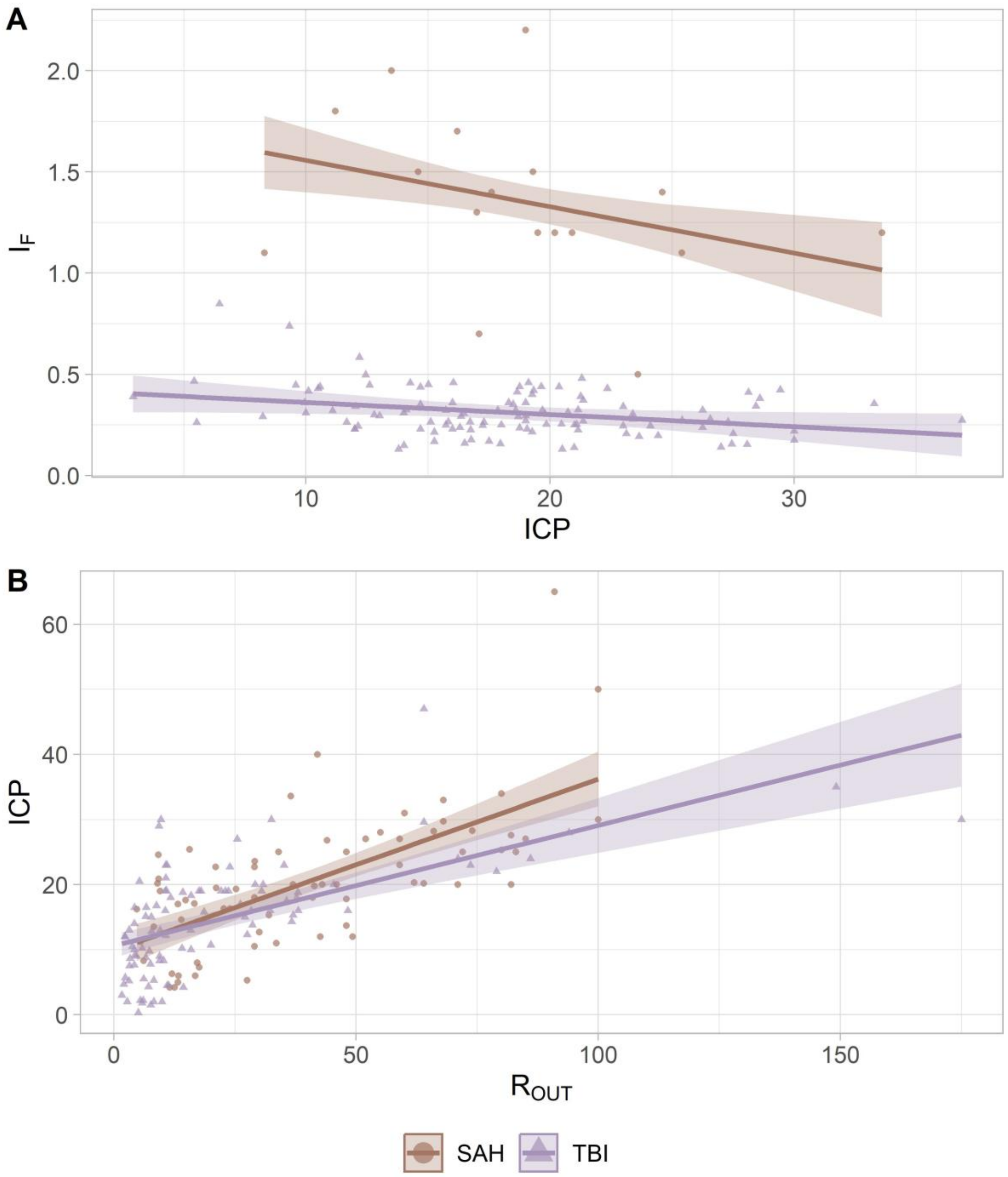

1. Alperin N, Bagci AM, Lee SH, Lam BL. Automated Quantitation of Spinal CSF Volume and Measurement of Craniospinal CSF Redistribution following Lumbar Withdrawal in Idiopathic Intracranial Hypertension. *AJNR Am J Neuroradiol*. Oct 2016;37(10):1957-1963. doi:https://dx.doi.org/10.3174/ajnr.A4837

2. Borgesen SE, Gjerris F. Relationships between intracranial pressure, ventricular size, and resistance to CSF outflow. Research Support, Non-U.S. Gov't. *Journal of Neurosurgery*. Oct 1987;67(4):535-9.

3. Cutler R, Page L, Galicich J, Watters G. Formation and absorption of cerebrospinal fluid in man. *Brain*. 1968;91(4):707-720.

4. Eide PK, Due-Tonnessen B, Helseth E, Lundar T. Assessment of intracranial pressure volume relationships in childhood: The lumbar infusion test versus intracranial pressure monitoring. *Child's Nervous System*. 2001;17(7):382-390. doi:https://dx.doi.org/10.1007/s003810000437

5. Eide PK, Fremming AD, Sorteberg A. Lack of relationship between resistance to cerebrospinal fluid outflow and intracranial pressure in normal pressure hydrocephalus. *Acta Neurol Scand*. December 2003;108(6):381-388. doi:https://dx.doi.org/10.1034/j.1600-0404.2003.00163.x

6. Gideon P, Sorensen PS, Thomsen C, Stahlberg F, Gjerris F, Henriksen O. Assessment of CSF dynamics and venous flow in the superior sagittal sinus by MRI in idiopathic intracranial hypertension: a preliminary study. Research Support, Non-U.S. Gov't. *Neuroradiology*. Jul 1994;36(5):350-4.

7. Gjerris F, Soelberg Sorensen P, Vorstrup S, Paulson OB. Intracranial pressure, conductance to cerebrospinal fluid outflow, and cerebral blood flow in patients with benign intracranial hypertension (pseudotumor cerebri). Research Support, Non-U.S. Gov't. *Annals of Neurology*. Feb 1985;17(2):158-62.

8. Gjerris F, Borgesen SE, Sorensen PS, et al. Resistance to cerebrospinal fluid outflow and intracranial pressure in patients with hydrocephalus after subarachnoid haemorrhage. Research Support, Non-U.S. Gov't. *Acta Neurochirurgica*. 1987;88(3-4):79-86.

9. Hansen K, Gjerris F, Sorensen PS. Absence of hydrocephalus in spite of impaired cerebrospinal fluid absorption and severe intracranial hypertension. Case Reports

Research Support, Non-U.S. Gov't. *Acta Neurochirurgica*. 1987;86(3-4):93-7.

10. Hayashi M, Kobayashi H, Fujii H, Yamamoto S. Ventricular size and isotope cisternography in patients with acute transient rises of intracranial pressure (plateau waves). Case Reports. *Journal of Neurosurgery*. Dec 1982;57(6):797-803.

11. Hayashi M, Handa Y, Kobayashi H, Kawano H, Ishii H, Hirose S. Plateau-wave phenomenon (I). Correlation between the appearance of plateau waves and CSF circulation in patients with intracranial hypertension. *Brain*. Dec 1991;114(Pt 6):2681-91.

12. Janny P, Chazal J, Colnet G, Irthum B, Georget AM. Benign intracranial hypertension and disorders of CSF absorption. Comparative Study. *Surgical Neurology*. Mar 1981;15(3):168-74.

13. Kosteljanetz M. CSF dynamics in patients with subarachnoid and/or intraventricular hemorrhage. *Journal of Neurosurgery*. May 1984;60(5):940-6.

14. Kosteljanetz M. Resistance to outflow of cerebrospinal fluid as a determinant of the intracranial pressure. Springer; 1986:123-127.

15. Kosteljanetz M. Acute head injury: pressure-volume relations and cerebrospinal fluid dynamics. *Neurosurgery*. Jan 1986;18(1):17-24.

16. Kosteljanetz M. CSF dynamics and pressure-volume relationships in communicating hydrocephalus. *Journal of Neurosurgery*. Jan 1986;64(1):45-52.

17. Lorenzo AV, Page LK, Watters GV. Relationship between cerebrospinal fluid formation, absorption and pressure in human hydrocephalus. *Brain*. 1970;93(4):679-92. doi:10.1093/brain/93.4.679

18. Marmarou A, Maset AL, Ward JD, et al. Contribution of CSF and vascular factors to elevation of ICP in severely head-injured patients. Research Support, Non-U.S. Gov't

Research Support, U.S. Gov't, P.H.S. *Journal of Neurosurgery*. Jun 1987;66(6):883-90.

19. Marmarou A, Foda MA, Bandoh K, et al. Posttraumatic ventriculomegaly: hydrocephalus or atrophy? A new approach for diagnosis using CSF dynamics. Research Support, U.S. Gov't, P.H.S. *Journal of Neurosurgery*. Dec 1996;85(6):1026-35.

20. Rubin RC, Henderson ES, Ommaya AK, Walker MD, Rall DP. The production of cerebrospinal fluid in man and its modification by acetazolamide. *Journal of neurosurgery*. 1966;25(4):430-436.

21. Sahuquillo J, Rubio E, Codina A, et al. Reappraisal of the intracranial pressure and cerebrospinal fluid dynamics in patients with the so-called Normal Pressure Hydrocephalus syndrome. *Acta Neurochirurgica*. 1991;112(1-2):50-61.

22. Shapiro K, Fried A, Marmarou A. Biomechanical and hydrodynamic characterization of the hydrocephalic infant. Research Support, Non-U.S. Gov't

Research Support, U.S. Gov't, P.H.S. *Journal of Neurosurgery*. Jul 1985;63(1):69-75.

23. Shapiro K, Fried A. Pressure-volume relationships in shunt-dependent childhood hydrocephalus. The zone of pressure instability in children with acute deterioration. Research Support, Non-U.S. Gov't

Research Support, U.S. Gov't, P.H.S. *Journal of Neurosurgery*. Mar 1986;64(3):390-6.

24. Silverberg GD, Huhn S, Jaffe RA, et al. Downregulation of cerebrospinal fluid production in patients with chronic hydrocephalus. Comparative Study

Research Support, Non-U.S. Gov't. *Journal of Neurosurgery*. Dec 2002;97(6):1271-5.

25. Stocchetti N, Bridelli F, Nizzoli V, Ravussin PA. Cerebral damage, fluid balance, intracranial pressure and pressure-volume relationship in subarachnoid haemorrhage. [French]. Hemorragie sous-arachnoidienne: Lesion cerebrale, equilibre hydrique, pression intracranienne et relation pression-volume. Conference Paper. *Annales Francaises d'Anesthesie et de Reanimation*. 1994;13(1):80-87.

Code

Ihsane Olakorede

Libraries

```r
library(broom)
library(broom.mixed)
library(DescTools)
library(dplyr)
library(ggplot2)
library(ggpubr)
library(ggResidpanel)
library(grid)
library(gridExtra)
library(gtsummary)
library(lme4)
library(lmerTest)
library(metafor)
library(orchaRd)
library(patchwork)
library(performance)
library(RColorBrewer)
library(rstatix)
library(tidyverse)
```

Functions

Forest Plot

```
compute_correlation <- function(subset_data) {

  n <- nrow(subset_data)

  correlation <- cor(subset_data$ICP, subset_data$I_f)

  standard_error <- FisherZInv(sqrt((1 - FisherZ(correlation)^2) / (n - 2)))

  sampling_variance <- standard_error^2

  return(c(n, correlation, standard_error, sampling_variance))
}

create_forest_plot <- function(correlation, res, psize, colz, colp, coll, k)
{
  par(mar=c(2.7,3.2,2.3,1.3), mgp=c(3,0,0), tcl=0.15)

  pred <- predict(res)

  if (pred$pi.lb < -1) {pred$pi.lb = -1.00}

  if (pred$pi.ub > 1) {pred$pi.ub = 1.00}

  sav <- forest(x = correlation$yi,
              vi = correlation$vi,
              ci.lb = correlation$ci_lb,
              ci.ub = correlation$ci_ub,
              ylim=c(-0.5,k+3),
              alim=c(-1,1),
              cex=1,
              pch=18,
              psize=psize,
              efac=0,
              refline=NA,
              lty=c(1,0),
              xlab="",
              slab=mapply(function(x,y) as.expression(bquote(.(x)^.(y))), paste("    ",
              ↪  correlation$Author, correlation$Year), correlation$Reference),
              rowadj=-.07,
              shade = "zebra2")

  segments(0, -1, 0, k+1.2, col=coll)

  segments(coef(res), 0, coef(res), k, col=colp, lty="33", lwd=0.8)

  segments(correlation$ci_lb, k:1, correlation$ci_ub, k:1, col=colz, lwd=1.75)

  points(correlation$yi, k:1, pch=18, cex=psize*1.2, col="white")
```

```
  points(correlation$yi, k:1, pch=18, cex=psize, col=colp)

  segments(pred$pi.lb, 0, pred$pi.ub, 0, col = "#A47764", lwd = 2, lty = 1)

  addpoly(res, row=0, mlab="", efac=2, col=colp, border=colp, cex=sav$cex)

  text(sav$xlim[1], -0.9, "Prediction Interval:", pos = 4, col = "#A47764")

  text(sav$xlim[2] - 0.6, -0.9, pos = 4, col = "#A47764",
       labels = sprintf("[%.2f, %.2f]", pred$pi.lb, pred$pi.ub))

  text(sav$xlim[1], 0, "Total Effect (95% CI)", pos = 4, font = 2)
  abline(h = k+1.2, col=coll)
  abline(h = 1, col=coll)
  axis(side=1, at=seq(-1,1,by=0.5), col=coll, labels=FALSE)

  par(xpd=NA)
  par(cex=sav$cex, font=2)
  text(sav$xlim[1], k+2, pos=4, "Study")
  text(0, k+2, "Correlation (95% CI)")
  text(sav$xlim[2]-0.3, k+2.2, "Correlation \n [95% CI]")
  text(sav$xlim[1], -2.2, pos=4, bquote(paste(I^2, " = ", .(round(res$I2)), "%", "; ",
                                        chi^2, "=", .(fmtx(res$QE, digits=2)), "; ",
                                        .(fmtp(res$QEp, digits=3, pname="P",
↪  add0=TRUE, equal=TRUE)))))

}
```

Plots - Figures

```
## Compute the Prediction and CI
predict_fun <- function(lm, data) {

  predslm = predict(lm, interval = "confidence")

  data <- cbind(data, predslm)

  return(data)

}

## Plot the Points with the Regression Line and CI
plot_fun <- function(data_orig, model, x, y, title, tag, x_label, y_label) {

  data <- predict_fun(model, data_orig)

  ggplot(data,
         aes(x = {{x}}, y = {{y}})) +
    geom_point(alpha = .7, color = "#A89A8F", size = 0.9) +
    geom_ribbon( aes(ymin = lwr, ymax = upr, color = NULL), fill = "#A47764", alpha = .3)
    ↪  +
```

```r
    geom_line( aes(y = fit), color = "#A47764", linewidth = 1) +
    theme_light() +
    labs(title = title, tag = tag, y = y_label, x = x_label) +
    theme(axis.text = element_text(size=10),
          axis.title = element_text(size=12),
          legend.text = element_text(size=10))

}

## Plot the Points with the Regression Line and CI (Groups)
plot_group_fun <- function(data_orig, model, x, y, group, title, tag, x_label, y_label,
↪   psize, lsize, gcolour, axis_size, x_size) {

  data <- predict_fun(model, data_orig)

  ggplot(data,
         aes(x = {{x}}, y = {{y}}, color = {{group}})) +
    geom_point(alpha = .7, show.legend = TRUE, size = psize) +
    geom_ribbon( aes(ymin = lwr, ymax = upr, fill = {{group}}, color = NULL), alpha = .3,
    ↪   show.legend = TRUE) +
    geom_line( aes(y = fit), linewidth = lsize, show.legend = TRUE) +
    theme_light() +
    scale_fill_manual(values = gcolour, drop = F) +
    scale_colour_manual(values = gcolour, drop = F) +
    labs(title = title, tag = tag, y = y_label, x = x_label) +
    theme(legend.position="top",
          legend.title = element_blank(),
          axis.text = element_text(size=axis_size),
          axis.title = element_text(size=x_size),
          legend.text = element_text(size=axis_size))

}
```

Analysis

```r
print_model_fun <- function(model) {

  model %>%
    tbl_regression(estimate_fun = function(x) style_number(x, digits = 2), intercept =
    ↪   TRUE) %>%
    add_glance_source_note(label = list(sigma ~ "\U03C3"),
                           include = c(r.squared, AIC, sigma))

}

t_test_oneway <- function(data, mu_value)
{
  print(shapiro.test(data))

  print(t.test(data, mu = mu_value, alternative = "two.sided"))
```

```
  print(wilcox.test(data, mu = mu_value, alternative = "two.sided"))

  print(median(data))
}
```

Main Pieces of Code

Forest Plot

```
## Color to be used in the plot
colpa <- "#6b58a6"

colp <- "#899F6A"

colz <- "#899F6A"

coll <- "#a7a9ac"

options(na.action = "na.pass")

## Load the original data
data_original <- read_csv("data.csv", na = "-") %>%
  mutate(Rcsf_method = as.character(Rcsf_method)) %>%
  mutate(If_method = as.character(If_method)) %>%
  mutate(Study = as.character(Study)) %>%
  filter(Population == "Human")

data_rout <- data_original %>%
 filter(!is.na(R_out))%>%
 filter(Study != 46)

data_if <- data_original %>%
 filter(!is.na(I_f))%>%
 filter(Study != 52)

## Compute the correlation and its standard error for each Study
correlation_if <- data_if %>%
 group_by(Study) %>%
 summarise(
   n = compute_correlation(cur_data())[1],
   correlation = compute_correlation(cur_data())[2],
   standard_error = compute_correlation(cur_data())[3],
   sampling_variance = compute_correlation(cur_data())[4]
 )

correlation_rout <- data_rout %>%
 group_by(Study) %>%
 summarise(
   n = compute_correlation(cur_data())[1],
   correlation = compute_correlation(cur_data())[2],
```

```r
  standard_error = compute_correlation(cur_data())[3],
  sampling_variance = compute_correlation(cur_data())[4]
 )

## Compute the effect size
correlation_if <- escalc(measure = "COR",
                      ri = correlation,
                      ni = n,
                      data = correlation_if)

correlation_rout <- escalc(measure = "COR",
                      ri = correlation,
                      ni = n,
                      data = correlation_rout)


## Define R_out parameters
res_rout <- rma(yi = yi, vi = vi, data = correlation_rout)

k_rout <- nrow(correlation_rout)

psize_k <- weights(res_rout)
psize_k <- 1.5 + (psize_k - min(psize_k)) / (max(psize_k) - min(psize_k))

## Define I_f parameters
res_if <- rma(yi = yi, vi = vi, data = correlation_if)

k_if <- nrow(correlation_if)

psize_i <- weights(res_if)
psize_i <- 1.5 + (psize_i - min(psize_i)) / (max(psize_i) - min(psize_i))
```

Analysis

```r
## Read the Data
data_original <- read_csv("data.csv", na = "-") %>%
  mutate(Rcsf_method = as.character(Rcsf_method)) %>%
  mutate(If_method = as.character(If_method)) %>%
  mutate(Study = as.character(Study))

## R_out - All

### Define Dataset
data_human_rout <- data_original %>%
  filter(Population == "Human") %>%
  filter(!is.na(R_out))%>%
  filter(Study != 46)

### Linear Model
lm_population <- lm(ICP ~ R_out,
                    data = data_human_rout)
```

```r
anova(lm_population)

print_model_fun(lm_population)

## R_out - Population

### Define Dataset
data_population_rout <- data_original %>%
  filter(Population == "Human") %>%
  filter(!is.na(R_out))%>%
  filter(Study != 46)

### Linear Model
lm_human_group <- lm(ICP ~ R_out * Population_explanation,
                     data = data_population_rout)

anova(lm_human_group)

print_model_fun(lm_human_group)

## R_out - Measurement Site

### Define Dataset
data_icpSite_rout <- data_original %>%
  filter(Population == "Human") %>%
  filter(!is.na(R_out))%>%
  filter(Study != 46)

data_icpSite_rout$ICP_site[data_icpSite_rout$ICP_site == "EVD"] <- "Cranial"

data_icpSite_rout$ICP_site[data_icpSite_rout$ICP_site == "Wire"] <- "Cranial"

### Linear Model
lm_icpSite <- lm(ICP ~ R_out * ICP_site,
                 data = data_icpSite_rout)

anova(lm_icpSite)

print_model_fun(lm_icpSite)

## R_out - Disease

### Define Dataset
data_disease_rout <- data_original %>%
  filter(Population == "Human") %>%
  filter(!Disease == "Mixed") %>%
  filter(!is.na(R_out))%>%
  filter(Study != 46)

data_disease_rout$Disease[data_disease_rout$Disease == "Atrophy" |
↪  data_disease_rout$Disease == "Parkinson" | data_disease_rout$Disease == "NPH"] <-
↪  "Neurodegenerative"
```

```r
data_disease_rout$Disease[data_disease_rout$Disease == "AVM" | data_disease_rout$Disease
↪  == "Brain Tumour"] <- "Brain Tumour"

data_disease_rout$Disease[data_disease_rout$Disease == "Craniosynostosis" |
↪  data_disease_rout$Disease == "HPH" | data_disease_rout$Disease == "Hydrocephalus" |
↪  data_disease_rout$Disease == "IIH"] <- "Primary Hydrocephalus"

data_disease_rout$Disease[data_disease_rout$Disease == "Meningitis" |
↪  data_disease_rout$Disease == "SAH" | data_disease_rout$Disease == "TBI"] <- "Acute
↪  Brain Injury"

data_disease_rout <- data_disease_rout %>%
  mutate(Disease = as.factor(Disease)) %>%
  filter(!Disease == "Brain Tumour")

### Linear Model
lm_disease <- lm(ICP ~ R_out * Disease,
                 data = data_disease_rout)

anova(lm_disease)

print_model_fun(lm_disease)

## R_out - Computation Method

### Define Dataset
data_formula_rout <- data_original %>%
  filter(Population == "Human") %>%
  filter(!is.na(R_out)) %>%
  filter(!is.na(Rcsf_method))%>%
  filter(Study != 46)

data_formula_rout$Rcsf_method[data_formula_rout$Rcsf_method == "1" |
↪  data_formula_rout$Rcsf_method == "3"] <- "Bolus"

data_formula_rout$Rcsf_method[data_formula_rout$Rcsf_method == "2" |
↪  data_formula_rout$Rcsf_method == "4"] <- "Infusion"

data_formula_rout$Rcsf_method[data_formula_rout$Rcsf_method == "5"] <- "Withdrawal"

### Linear Model
lm_formula <- lm(R_out ~ ICP * Rcsf_method,
                 data = data_formula_rout)

anova(lm_formula)

print_model_fun(lm_formula)

## R_out - Disease2

### Define Dataset
data_disease2_rout <- data_original %>%
```

```
  filter(Population == "Human") %>%
  filter(!Disease == "Mixed") %>%
  filter(!Disease == "AVM") %>%
  filter(!Disease == "Brain Tumour") %>%
  filter(!is.na(R_out))%>%
  filter(Study != 46)

data_disease2_rout$Disease[data_disease2_rout$Disease == "Atrophy" |
↪  data_disease2_rout$Disease == "Parkinson" | data_disease2_rout$Disease == "NPH"] <-
↪  "Neurodegenerative"

data_disease2_rout$Disease[data_disease2_rout$Disease == "Craniosynostosis" |
↪  data_disease2_rout$Disease == "HPH" | data_disease2_rout$Disease == "Hydrocephalus" |
↪  data_disease2_rout$Disease == "IIH"] <- "Primary Hydrocephalus"

data_disease2_rout$Disease[data_disease2_rout$Disease == "Meningitis" |
↪  data_disease2_rout$Disease == "SAH" | data_disease2_rout$Disease == "TBI"] <- "Acute
↪  Brain Injury"

### Linear Model
lm_disease2 <- lm(R_out ~ ICP * Disease,
                  data = data_disease2_rout)

anova(lm_disease2)

print_model_fun(lm_disease2)

## I_f - All

### Define Dataset
data_human_if <- data_original %>%
  filter(Population == "Human") %>%
  filter(!is.na(I_f)) %>%
  filter(!Study == 52)

### Linear Model
lm_population_i <- lm(I_f ~ ICP,
                      data = data_human_if)

anova(lm_population_i)

print_model_fun(lm_population_i)

t_test_oneway(data_human_if$I_f, 0.28)

## I_f - Population

### Define Dataset
data_population_if <- data_original %>%
  filter(Population == "Human") %>%
  filter(!Population_explanation == "Mixed") %>%
  filter(!is.na(I_f))%>%
  filter(!Study == 52)
```

```r
### Linear Model
lm_human_group_i <- lm(I_f ~ ICP * Population_explanation,
                      data = data_population_if)

anova(lm_human_group_i)

print_model_fun(lm_human_group_i)

t_test_oneway(data_population_if[data_population_if$Population_explanation ==
↪  "Adult",]$I_f, 0.3)

t_test_oneway(data_population_if[data_population_if$Population_explanation ==
↪  "Paediatric",]$I_f, 0.22)

## I_f - Measurement Site

### Define Dataset
data_icpSite_if <- data_original %>%
  filter(Population == "Human") %>%
  filter(!is.na(I_f))%>%
  filter(!Study == 52)

data_icpSite_if$ICP_site[data_icpSite_if$ICP_site == "EVD"] <- "Cranial"

data_icpSite_if$ICP_site[data_icpSite_if$ICP_site == "Wire"] <- "Cranial"

### Linear Model
lm_icpSite_i <- lm(I_f ~ ICP * ICP_site,
                  data = data_icpSite_if)

anova(lm_icpSite_i)

print_model_fun(lm_icpSite_i)

t_test_oneway(data_icpSite_if[data_icpSite_if$ICP_site == "Cranial",]$I_f, 0.29)

t_test_oneway(data_icpSite_if[data_icpSite_if$ICP_site == "Lumbar",]$I_f, 0.4)

## I_f - Disease

### Define Dataset
data_disease_if <- data_original %>%
  filter(Population == "Human") %>%
  filter(!Disease == "Mixed") %>%
  filter(!is.na(I_f)) %>%
  filter(!Study == 52)

data_disease_if$Disease[data_disease_if$Disease == "Atrophy" | data_disease_if$Disease ==
↪  "Parkinson" | data_disease_if$Disease == "NPH"] <- "Neurodegenerative"

data_disease_if$Disease[data_disease_if$Disease == "AVM" | data_disease_if$Disease ==
↪  "Brain Tumour"] <- "Brain Tumour"
```

```r
data_disease_if$Disease[data_disease_if$Disease == "Craniosynostosis" |
↪  data_disease_if$Disease == "HPH" | data_disease_if$Disease == "Hydrocephalus" |
↪  data_disease_if$Disease == "IIH"] <- "Primary Hydrocephalus"

data_disease_if$Disease[data_disease_if$Disease == "Meningitis" | data_disease_if$Disease
↪  == "SAH" | data_disease_if$Disease == "TBI"] <- "Acute Brain Injury"

data_disease_if <- data_disease_if %>%
  mutate(Disease = as.factor(Disease)) %>%
  filter(!Disease == "Neurodegenerative")

### Linear Model
lm_disease_i <- lm(I_f ~ ICP * Disease,
                 data = data_disease_if)

anova(lm_disease_i)

print_model_fun(lm_disease_i)

t_test_oneway(data_disease_if[data_disease_if$Disease == "Acute Brain Injury",]$I_f,
↪  0.28)

t_test_oneway(data_disease_if[data_disease_if$Disease == "Primary Hydrocephalus",]$I_f,
↪  0.29)

## R_out - TBI vs SAH

### Define Dataset
data_tbisah_rout <- data_original %>%
  filter(Population == "Human") %>%
  filter(Disease == "TBI" | Disease == "SAH") %>%
  filter(!is.na(R_out))

### Linear Model
lm_tbisah <- lm(ICP ~ R_out * Disease,
                data = data_tbisah_rout)

anova(lm_tbisah)

print_model_fun(lm_tbisah)

## I_f - TBI vs SAH

### Define Dataset
data_tbisah_if <- data_original %>%
  filter(Population == "Human") %>%
  filter(Disease == "TBI" | Disease == "SAH") %>%
  filter(!is.na(I_f)) %>%
  filter(!Study == 52)

### Linear Model
lm_tbisah_i <- lm(I_f ~ ICP * Disease,
```

```
                  data = data_tbisah_if)

anova(lm_tbisah_i)

print_model_fun(lm_tbisah_i)

t_test_oneway(data_tbisah_if[data_tbisah_if$Disease == "TBI",]$I_f, 0.26)

t_test_oneway(data_tbisah_if[data_tbisah_if$Disease == "SAH",]$I_f, 0.18)

## R_out - Complete model

### Define Dataset

data_complete_rout <- data_original %>%
  filter(Population == "Human") %>%
  filter(!is.na(R_out))%>%
  filter(Study != 46)

#### ICP Site
data_complete_rout$ICP_site[data_complete_rout$ICP_site == "EVD"] <- "Cranial"

data_complete_rout$ICP_site[data_complete_rout$ICP_site == "Wire"] <- "Cranial"

#### Disease
data_complete_rout$Disease[data_complete_rout$Disease == "Atrophy" |
↪  data_complete_rout$Disease == "Parkinson" | data_complete_rout$Disease == "NPH"] <-
↪  "Neurodegenerative"

data_complete_rout$Disease[data_complete_rout$Disease == "AVM" |
↪  data_complete_rout$Disease == "Brain Tumour"] <- "Brain Tumour"

data_complete_rout$Disease[data_complete_rout$Disease == "Craniosynostosis" |
↪  data_complete_rout$Disease == "HPH" | data_complete_rout$Disease == "Hydrocephalus" |
↪  data_complete_rout$Disease == "IIH"] <- "Primary Hydrocephalus"

data_complete_rout$Disease[data_complete_rout$Disease == "Meningitis" |
↪  data_complete_rout$Disease == "SAH" | data_complete_rout$Disease == "TBI"] <- "Acute
↪  Brain Injury"

#### Computation Method
data_complete_rout$Rcsf_method[data_complete_rout$Rcsf_method == "1" |
↪  data_complete_rout$Rcsf_method == "3"] <- "Bolus"

data_complete_rout$Rcsf_method[data_complete_rout$Rcsf_method == "2" |
↪  data_complete_rout$Rcsf_method == "4"] <- "Infusion"

data_complete_rout$Rcsf_method[data_complete_rout$Rcsf_method == "5"] <- "Withdrawal"

### Linear Model

#### Without Random Effect
lm_complete <- lm(ICP ~ R_out + Population_explanation + ICP_site + Disease,
```

```r
                    data = data_complete_rout)

anova(lm_complete)

print_model_fun(lm_complete)

#### With Random Effect

lme_complete <- lmer(ICP ~ R_out + Population_explanation + ICP_site + Disease +
↪   (1|Study),
                       data = data_complete_rout)

summary(lme_complete)

anova(lme_complete)

anova(lme_complete, lm_complete)

## I_f - Complete model

### Define Dataset

data_complete_if <- data_original %>%
  filter(Population == "Human") %>%
  filter(!is.na(I_f))%>%
  filter(Study != 52)

#### ICP Site
data_complete_if$ICP_site[data_complete_if$ICP_site == "EVD"] <- "Cranial"

data_complete_if$ICP_site[data_complete_if$ICP_site == "Wire"] <- "Cranial"

#### Disease
data_complete_if$Disease[data_complete_if$Disease == "Atrophy" | data_complete_if$Disease
↪   == "Parkinson" | data_complete_if$Disease == "NPH"] <- "Neurodegenerative"

data_complete_if$Disease[data_complete_if$Disease == "AVM" | data_complete_if$Disease ==
↪   "Brain Tumour"] <- "Brain Tumour"

data_complete_if$Disease[data_complete_if$Disease == "Craniosynostosis" |
↪   data_complete_if$Disease == "HPH" | data_complete_if$Disease == "Hydrocephalus" |
↪   data_complete_if$Disease == "IIH"] <- "Primary Hydrocephalus"

data_complete_if$Disease[data_complete_if$Disease == "Meningitis" |
↪   data_complete_if$Disease == "SAH" | data_complete_if$Disease == "TBI"] <- "Acute
↪   Brain Injury"

#### Computation Method

data_complete_if$If_method[data_complete_if$If_method == "1" ] <- "Method 1"

data_complete_if$If_method[data_complete_if$If_method == "2" ] <- "Method 2"
```

```r
data_complete_if$If_method[data_complete_if$If_method == "3" ] <- "Method 3"


### Linear Model

#### Without Random Effect
lm_complete_i <- lm(I_f ~ ICP + Population_explanation + ICP_site + Disease,
                  data = data_complete_if)

anova(lm_complete_i)

print_model_fun(lm_complete_i)

#### With Random Effect

lme_complete_i <- lmer(I_f ~ ICP + Population_explanation + ICP_site + Disease +
↪  (1|Study),
                     data = data_complete_if)

summary(lme_complete_i)

anova(lme_complete_i)

anova(lme_complete_i, lm_complete_i)
```

Plots - Figures

```r
## Color to be used in the plot
g_population <- c("#D19C97", "#7391C9")

g_disease <- c("#899F6A", "#7391C9", "#A47764", "#A793B9")

g_site <- c("#A89A8F", "#899F6A")

g_method <- c("#D19C97", "#A793B9", "#A47764")

g_st <- c("#A47764", "#A793B9")

## Single Plots
rout_all_patients <- plot_fun(data_human_rout, lm_population, R_out, ICP, NULL, NULL,
↪  expression(paste(R[OUT])), "ICP")

rout_population <- plot_group_fun(data_population_rout, lm_human_group, R_out, ICP,
↪  Population_explanation, NULL, NULL, expression(paste(R[OUT])), "ICP",
                                  0.9, 1, g_population, 10,12)

rout_measurement_site <- plot_group_fun(data_icpSite_rout, lm_icpSite, R_out, ICP,
↪  ICP_site, NULL, NULL, expression(paste(R[OUT])), "ICP",
                                        0.9, 1, g_site, 10,12)

rout_disease <- plot_group_fun(data_disease_rout, lm_disease, R_out, ICP, Disease, NULL,
↪  NULL, expression(paste(R[OUT])), "ICP",
```

```
                                0.9, 1, g_disease, 10,12)

rout_computation_methods <- plot_group_fun(data_formula_rout, lm_formula, ICP, R_out,
↪  Rcsf_method, NULL, NULL, "ICP", expression(paste(R[OUT])),
                                            0.9, 1, g_method, 10,12)

rout_disease2 <- plot_group_fun(data_disease2_rout, lm_disease2, ICP, R_out, Disease,
↪  NULL, NULL, "ICP", expression(paste(R[OUT])),
                                 0.9, 1, c("#899F6A", "#A47764", "#A793B9"), 10,12)

if_all_patients <- plot_fun(data_human_if, lm_population_i, ICP, I_f, NULL, NULL, "ICP",
↪  expression(paste(I[F])))

if_population <- plot_group_fun(data_population_if, lm_human_group_i, ICP, I_f,
↪  Population_explanation, NULL, NULL, "ICP", expression(paste(I[F])),
                                 0.9, 1, g_population, 10,12)

if_measurement_site <- plot_group_fun(data_icpSite_if, lm_icpSite_i, ICP, I_f, ICP_site,
↪  NULL, NULL, "ICP", expression(paste(I[F])),
                                       0.9, 1, g_site, 10,12)

if_disease <- plot_group_fun(data_disease_if, lm_disease_i, ICP, I_f, Disease, NULL,
↪  NULL, "ICP", expression(paste(I[F])),
                              0.9, 1, g_disease, 10,12)

rout_sahtbi <- plot_group_fun(data_tbisah_rout, lm_tbisah, R_out, ICP, Disease, NULL,
↪  NULL, expression(paste(R[OUT])), "ICP",
                               0.9, 1, g_st, 10,12)

if_sahtbi <- plot_group_fun(data_tbisah_if, lm_tbisah_i, ICP, I_f, Disease, NULL, NULL,
↪  "ICP", expression(paste(I[F])),
                             0.9, 1, g_st, 10,12)
```

Create Figures

Forest Plots

```
## I_f Forest Plot
jpeg("Forest_if.jpg", width = 8*600, height = 6*600, res = 600)
create_forest_plot(correlation_if, res_if, psize_i, colz, colp, coll, k_if)
dev.off()

## R_out Forest Plot
jpeg("Forest_rout.jpg", width = 8*600, height = 6*600, res = 600)
create_forest_plot(correlation_rout, res_rout, psize_k, colz, colp, coll, k_rout)
dev.off()
```

Plots - Figures

```
## Figure 3 - All points
jpeg("Figure_3.jpeg", width = 6*600, height = 7*600, res = 600)
ggarrange(if_all_patients, rout_all_patients,
                      labels = c("A", "B"),
                      font.label = list(size = 13),
                      ncol = 1, nrow = 2, align = "hv")
dev.off()

## Figure 4 - Population, Disease, ICP Measurement site
jpeg("Figure_4.jpeg", width = 7*600, height = 8*600, res = 600)
ggarrange(ggarrange(if_population, rout_population, ncol = 2, labels = c("A", "D"),
↪  font.label = list(size = 13), common.legend = TRUE, legend="bottom"),
                      ggarrange(if_disease, rout_disease, ncol = 2, labels = c("B", "E"),
                      ↪  font.label = list(size = 13), common.legend = TRUE,
                      ↪  legend="bottom"),
                      ggarrange(if_measurement_site, rout_measurement_site, ncol = 2,
                      ↪  labels = c("C", "F"), font.label = list(size = 13),
                      ↪  common.legend = TRUE, legend="bottom"),
                      ncol = 1, nrow = 3, align = "hv")
dev.off()

## Figure 5 - R_out computation method, Disease v2
jpeg("Figure_5.jpeg", width = 6*600, height = 7*600, res = 600)
ggarrange(rout_computation_methods, rout_disease2,
                      labels = c("A", "B"),
                      font.label = list(size = 13),
                      legend = "bottom",
                      ncol = 1, nrow = 2, align = "hv")
dev.off()

## Figure Supplement 2 - SAH vs TBI
jpeg("Figure_supp_2.jpeg", width = 6*600, height = 7*600, res = 600)
ggarrange(if_sahtbi, rout_sahtbi,
                      ncol = 1, nrow = 2, labels = c("A", "B"),
                      font.label = list(size = 13),
                      common.legend = TRUE, legend="bottom")
dev.off()
```
